# Connecting complex and simplified models of tipping elements: a nonlinear two-forcing emulator for the Atlantic meridional overturning circulation

**DOI:** 10.12688/openreseurope.19479.1

**Published:** 2025-03-31

**Authors:** Amaury Laridon, Victor Couplet, Justin Gérard, Wim Thiery, Michel Crucifix

**Affiliations:** 1 Water and Climate, Vrije Universiteit Brussel, Brussels, Brussels, 1050, Belgium; 2 Earth and Life Institute, Universite catholique de Louvain, Louvain-la-Neuve, Walloon Region, 1348, Belgium

**Keywords:** Climate change, Tipping Points, AMOC, Emulator, Non-linear dynamics, SURFER, cGENIE.

## Abstract

**Background:**

Despite its far-reaching implications, accurately characterizing the tipping dynamics of the Atlantic Meridional Overturning Circulation (AMOC) remains a significant challenge. Complex models, including Earth System Models (ESMs) and Earth System Models of Intermediate Complexity (EMICs), offer valuable insights; however, they are computationally expensive and subject to substantial uncertainties in identifying AMOC tipping points. In contrast, simple conceptual models based on non-linear dynamics have been developed to represent tipping elements such as the AMOC. These models can be calibrated against complex models to explore various scenarios and forcing spaces, functioning as emulators. Traditionally, such emulators have focused on a single forcing variable, typically global mean temperature, despite the well-established influence of freshwater fluxes on AMOC dynamics. Moreover, existing two-forcing AMOC emulators lack robust calibration methods against complex models.

**Methods:**

In this study, we develop and validate a two-forcing AMOC emulator that incorporates global mean temperature and freshwater flux, grounded in non-linear dynamics. The emulator is calibrated against the AMOC response within the EMIC cGENIE. After validation, the emulator is integrated into SURFER, a reduced-complexity climate model, enabling rapid simulation of AMOC trajectories under diverse emission scenarios.

**Results:**

By considering Greenland Ice Sheet melt, the emulator captures two additional collapses and one overshoot without tipping trajectories for emission scenarios ranging from SSP3-7.0 to SSP5-8.5. Furthermore, the emulator allows the assessment of the critical forcing manifold of the AMOC in the complex model, enabling the identification of combined forcing thresholds for the AMOC and serving as a tool for comparing the sensitivities of complex models.

**Conclusions:**

With its low computational cost and calibration accuracy, our tool represents a significant advancement in exploring AMOC dynamics in future climatic scenarios. Finally, the methodology used to develop this emulator is generalizable, providing a framework for studying other tipping elements in research.

## Plain language summary

As global temperatures rise due to greenhouse gas emissions, certain key components of the Earth’s climate system are approaching critical thresholds known as tipping points, beyond which significant changes may occur. One such tipping element is the Atlantic Meridional Overturning Circulation (AMOC), a crucial ocean current responsible for redistributing heat between the hemispheres. If the AMOC were to collapse, it could result in substantial regional changes in temperature, precipitation, and other critical aspects of the climate. However, the exact location of the AMOC tipping point and the time it would take to collapse remain uncertain due to limitations in complex climate models. Additionally, running these complex models is computationally expensive, making it difficult to explore a wide range of potential future scenarios. In this study, we developed an emulator—a simplified conceptual model calibrated using a complex model—to reproduce the behaviour of the AMOC during a potential collapse. This emulator is built on a new methodology that improves the alignment between simplified dynamics and complex models. The resulting tool enables the production of numerous AMOC simulations under various emission scenarios with low computational cost, while remaining consistent with our understanding of the physical processes that govern the AMOC. For example, this new emulator simulate AMOC collapse under future high emission pathways. This emulator and methods enables researchers to better explore the potential responses of the AMOC to future emissions and strengthens our ability to predict and prepare for abrupt changes in the climate system.

## 1. Introduction

### 1.1 The AMOC as a tipping element: Addressing high uncertainties

The Atlantic Meridional Overturning Circulation (AMOC) is a key component of the climate system. It plays a central role in the transport of heat and salt throughout the global ocean and significantly influences both regional and global climate dynamics ^1– 3^. The AMOC has been identified as a tipping element, a large-scale component of the climate system that can reach a tipping point ^4^. A tipping point refers to a critical threshold in a forcing parameter, known as the bifurcation parameter, beyond which a small perturbation of this parameter can cause the tipping element to transition from one equilibrium state to another, resulting in a significant qualitative change ^4^. For the AMOC, the secondary stable equilibrium corresponds to a collapse state, where the circulation ceases ^5^. A cessation or even a slowdown of the AMOC would have significant consequences for temperatures in the North Atlantic ^3– 6^, as well as impacts on the carbon cycle ^7^, monsoons ^2^, and, potentially, other tipping elements ^5, 8, 9^.

Effectively simulating the AMOC requires an understanding of its physical dynamics. The AMOC operates through the sinking of large amounts of warm, salty water from the South Atlantic into the North Atlantic. As this water cools, it becomes sufficiently dense to sink into the depths, forming the North Atlantic Deep Water (NADW), which then returns to the South Atlantic ^10, 11^. The AMOC is a component of what is referred to as the thermohaline circulation, as it is driven by density differences determined by the temperature and salinity of the water. The tipping point of the AMOC corresponds to a critical threshold in the stratification of Atlantic waters. With global warming, the increase in Atlantic water temperature reduces its density, thereby enhancing its buoyancy ^12^. This thermal forcing is the dominant mechanism driving AMOC weakening ^13– 15^. The second forcing mechanism involves a disturbance in the salinity of the water within the Atlantic. As the Greenland Ice Sheet (GIS) melts due to global warming, large quantities of freshwater are added to the deep-water sinking regions in the North Atlantic. This freshwater flux reduces the water’s density, increasing its buoyancy and diminishing its ability to sink into the depths, thereby weakening the intensity of the AMOC ^16– 18^. On the other hand, future variations in water salinity in the North Atlantic may result from changes in the precipitation-evaporation (P-E) balance driven by temperature anomalies ^19^. Consequently, there exists a critical threshold of water stratification that, if exceeded for a prolonged period, can become self-sustaining, as the AMOC can no longer transport warm and salty water to the North Atlantic ^20^, ultimately leading to the collapse of the circulation ^5, 16, 21^. Once reached, this collapse state is irreversible, as returning to the critical value of the bifurcation parameter will not allow the system to return to its initial equilibrium. A characteristic hysteresis phenomenon is thus present, preventing a return to the initial state within timescales relevant to human lifetimes ^4, 22^.

There is evidence that the AMOC has slowed during the 20 ^th^ century ^23^, with reconstructions indicating a 15% decline over the past 70 years ^24^, bringing it closer to its tipping point. However, observations of AMOC slowdown are subject to considerable uncertainty ^10^, and although the AMOC may have collapsed in the past ^8, 25, 26^, accurately forecasting its future evolution—when and at what rate it may collapse remains a significant challenge ^5, 27, 28^. Estimates of the tipping point for the AMOC must therefore rely on models, but the results vary significantly ^28^. Some studies place it at a global mean temperature anomaly of 8°C, while others suggest it may already be as low as 1.4°C ^29^. Consequently, some studies estimate that a complete collapse of the AMOC could occur by the end of this century ^30^, or not until 2300 ^16^. In addition, there is also sensitivity to the rate of the applied forcing ^31, 32^. Lastly, the tipping dynamics of the AMOC cannot be fully understood without considering those of the GIS, which has a melting threshold beyond which its decline becomes irreversible ^33^. In addition to the destabilizing effect of GIS melting on the AMOC due to freshwater input, a collapse of the AMOC would, conversely, have a stabilizing effect on the GIS. Indeed, the resulting regional cooling following an AMOC shutdown would slow down the melting of the GIS. Thus, a coupling between the AMOC and the GIS exists, potentially giving rise to tipping cascades ^34^, where the collapse of one system triggers the failure of another or, conversely, helps stabilize it ^35^. However, significant uncertainties remain in the projections of these dynamics ^5^.

### 1.2 The need for simplified models capturing first-order dynamics

This significant uncertainty regarding the future evolution of AMOC collapse stems from the diversity of models and approaches employed. To assess the stability of the AMOC and the associated thresholds, stability diagrams are constructed using hysteresis experiments, in which the forcing is slowly varied along a back-and-forth scenario. However, due to their prohibitive computational demands, complex models, such as Earth System Models (ESMs) are unable to simulate numerous complete AMOC hysteresis curves ^36^. Consequently, Earth System Models of Intermediate Complexity (EMICs) offer the best compromise between explicitly resolving processes and computational efficiency for generating AMOC hysteresis curves ^27, 37^. However, even EMICs exhibit significant variability in the location of the tipping point, and consequently, in their projections of the future evolution of the AMOC ^27^. Moreover, due to their significant computational costs compared to simpler models, these complex models are not well-suited for efficiently exploring the range of potential forcing scenarios and their associated AMOC responses. Additionally, there is evidence that the current generation of complex climate models exhibits excessive stability due to biases in ocean salinity distribution ^38, 39^. Consequently, recent studies have increasingly relied on conceptual models to simulate the AMOC and other tipping elements ^35, 40– 42^.

This approach primarily relies on modelling the dynamics of tipping elements through a double-fold bifurcation structure ^40– 43^, as stability indicators and observational analyses have shown that the AMOC resides in a bi-stable regime ^44– 46^. In this framework, the system possesses two stable equilibria separated by an unstable equilibrium. When the bifurcation parameter reaches the critical value of the tipping point, the system can transition from its initial stable equilibrium to the second. These models mathematically impose that the AMOC behaves as a nonlinear dynamical system with this specific double-fold structure, thereby defining its intrinsic tipping element dynamics. The primary advantage of these models, beyond their ability to capture the suspected dynamical behaviour, is their computational efficiency. By calibrating these simplified dynamics using experiments from complex models, these models enable the creation of tipping element emulators. When these tipping element emulators are coupled with a reduced-complexity climate model, they offer a tool that is both process-based and computationally efficient. This enables the study of tipping element evolution in multi-millennial simulations under realistic emissions scenarios ^40^.

### 1.3 Challenges in designing a two-forcing emulator

An emulator based on the concept of a double-fold bifurcation was introduced by Martinez Montero *et al*. ^47^ in the reduced-complexity climate model SURFER v2.0 to simulate the dynamics of ice sheets. This emulator considers only a single forcing variable, specifically the temperature anomaly computed from emission scenarios. In SURFER v3.0, Couplet *et al*. ^40, 48^ developed a generalization of this emulator, allowing for the coupling of tipping elements and hence the inclusion of an additional forcing variable. The challenge, however, lies in establishing a calibration technique for the model parameters based on existing literature and complex models. In their study, Couplet *et al*. ^40^ employed a Monte Carlo sampling method within the parameter space of their simplified models to reproduce the configurations considered plausible in the literature regarding the state of tipping elements. In contrast to Couplet *et al*. ^40^, our approach focuses on calibrating the parameters of a two-parameter physical forcing emulator to align with any given hysteresis curve from a complex model. This methodology enables a more realistic calibration of the emulator while facilitating the systematic exploration of the forcing space across different emission scenarios over extended timescales. Importantly, it preserves the key characteristics of the emulated complex model, allowing for computationally efficient yet physically consistent simulations.

To our knowledge, no simplified tipping element model with two forcing parameters, capable of being calibrated using experiments from complex models, has been developed. However, the AMOC is influenced by two forcing variables: temperature anomaly and freshwater flux. Given the necessity of more realistically constraining potential future AMOC trajectories, this study seeks to address the following objective: develop an AMOC emulator with two forcing parameters — temperature and freshwater flux — that can be calibrated against hysteresis from complex models and integrated into a climate model. The paper is structured as follows. In Section 2, we introduce the AMOC Tipping Calibration Module (ATCM), a simplified nonlinear dynamical model based on the double-fold bifurcation structure with two forcing parameters: temperature anomaly and freshwater flux. We also describe the calibration module used to fit the double-fold structure to any hysteresis curve derived from a complex model. This novel module is based on the assumption of independent forcing calibration experiments, which allows for a generalization of the method by Martinez Monteiro *et al*. ^47^. In Section 3, we apply this calibration process to cGENIE, an EMIC, using two calibration experiments. We then validate the ATCM as an emulator by comparing its results with those of one additional cGENIE experiment. Subsequently, we integrate the ATCM into the SURFER climate model, creating a new configuration that provides a calibrated emulator of the AMOC within a fast climate model. This setup enables the analysis of overshoot without tipping phenomena. Furthermore, it facilitates further investigations into the potential collapse dynamics of the AMOC under realistic emission scenarios, utilizing the critical manifold of the complex model's sensitivity, which can be captured by the emulator. Section 4 presents an analysis of our key findings, comparing them with previous studies. It also discusses the limitations of our approach in emulating a complex dynamic using a conceptual non-linear dynamics model and outlines potential improvements to this methodology, as well as its application to emulate other tipping elements. Finally, we conclude in Section 5.

## 2. Methods

We introduce the AMOC Tipping Calibration Module (ATCM), an emulator designed to represent the dynamics of the AMOC tipping element with two forcing parameters: temperature and freshwater flux. The key feature of this emulator is its ability to calibrate simplified AMOC dynamics to match any hysteresis curve derived from a more complex, process-based model. We detail the equations that define the model and describe the methodology used for its calibration. The AMOC model is based on the normal form of a double-fold bifurcation, which has been demonstrated in previous studies to be effective in modelling the tipping element behaviour of the AMOC ^40, 42^. The calibration technique builds upon the ice sheet emulator implemented in SURFER v2.0 ^47^, incorporating the assumption of independent forcing in the calibration experiments. Subsequently, in the results section, the ATCM is calibrated on the cGENIE model, enabling it to act as an emulator for the AMOC.

### 2.1 AMOC dynamical model

**Figure 1.**
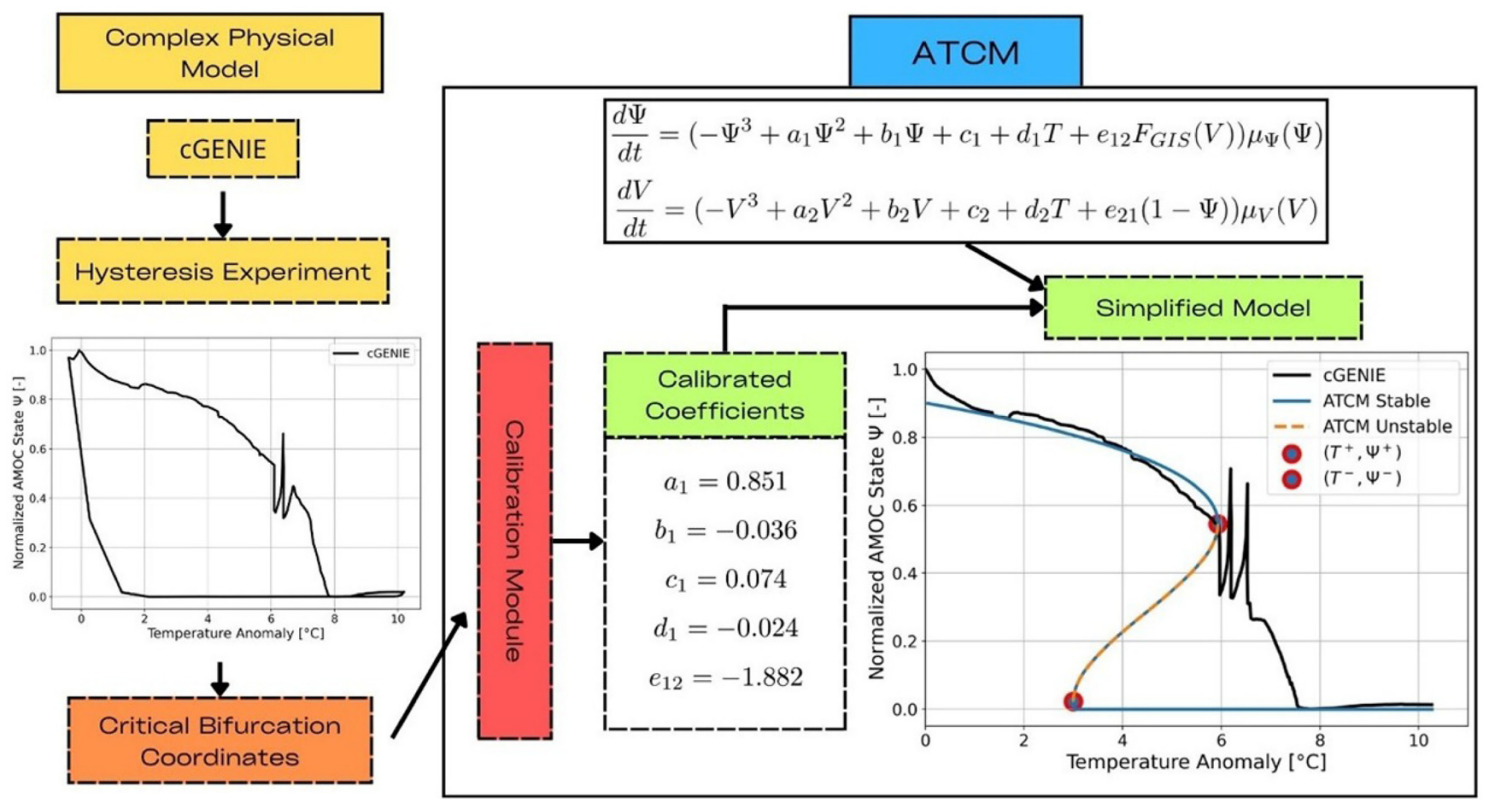
Schematic of the AMOC Tipping Calibration Module (ATCM). The module requires input in the form of bifurcation coordinates derived from complex experiments. These coordinates are used to adjust the calibration coefficients within the simplified tipping element model.

The overall scheme of the ATCM is depicted in Figure 1. Given that one of the primary freshwater flux forcings of the AMOC is GIS melting, and that we aim to develop a framework that can subsequently be used to study tipping cascade phenomena, we also provide an explicit mathematical formulation of the GIS model. The non linear ordinary differential equations that describes the AMOC intensity and the GIS volume are written as,

$$\frac{\text{d}\Psi }{\text{dt}}\left(\text{Ψ},\text{T},{\text{F}}_{\text{GIS}}\right)=\left(\overset{\text{internal}\;\text{dynamics}}{\overset{︷}{-{\text{Ψ}}^{3}+{\text{a}}_{1}{\text{Ψ}}^{2}+{\text{b}}_{1}\text{Ψ}+{\text{c}}_{1}}}+\overset{\text{forcings}}{\overset{︷}{{\text{d}}_{1}\text{T}+{\text{e}}_{12}{\text{F}}_{\text{GIS}}}}\right)\mu_{\Psi }\left(\text{Ψ}\right),\;(1)$$

$$\frac{dV}{dt}(V,T,\text{Ψ})=\left(\underset{\text{internal}\;\text{dynamics}}{\underset{︸}{-V^{3}+a_{2}V^{2}+b_{2}V+c_{2}}}+\underset{\text{focings}}{\underset{︸}{d_{2}T+e_{21}(1-\text{Ψ})}}\right)\mu_{V}(V).\;(2)$$

These equations are derived from the normal form of a double-fold bifurcation, enabling the representation of the first-order tipping dynamics of the AMOC and the GIS. Consequently, the tipping element model acts as a generic dynamical system designed to function as an emulator. The use of a generalized normal form of a double-fold bifurcation has already been demonstrated to be effective for tipping element models ^40, 42, 47^. The state variables *Ψ* and *V* are dimensionless variables and span the interval [0,1], defining the state of the AMOC and the GIS respectively compared to their pre-industrial state. For the AMOC, *Ψ* is the ratio of its current intensity in *Sv* with its pre-industrial value while for the GIS, *V* is the ratio between the ice volume and its pre-industrial value. The first group of terms in Equation 1 and Equation 2 represents the internal dynamics of the tipping element. Since it is a cubic polynomial of the state variable it allows the tipping element to have 1,2 or 3 stable states depending on its forcings ^40^. The coefficients *a _i_,b _i_,c _i_,d _i_,e _ij_* ( *i,j* = 1,2, *i* ≠ *j*) do not correspond immediately to specific physical properties, but by setting these parameters, one can reproduce the stability structure diagnosed in more complex models.

The first forcing term, *T*, which appears in Equation (1) and Equation (2) represents the global mean surface air temperature anomaly relative to pre-industrial levels. In Equation (1), this term encapsulates the physical effects of warming on AMOC water stratification, as well as freshwater anomalies resulting from simulated P-E in the complex model. In Equation (2) the *T* forcing term represents the impact of temperature on ice melting in the GIS. In Equation (1), the second forcing term, *F _GIS_*, represents the freshwater flux resulting from GIS melting, which weakens the intensity of the AMOC. The parameterization of *F _GIS_* is based on that of Couplet *et al*. ^40^ and is defined as follow:

$$\;F_{GIS}=\alpha_{GIS}\frac{dV}{dt}.\;(3)$$

The freshwater contribution of the GIS is proportional to the temporal variation of its dimensionless volume. The parameter *α _GIS_* relates the temporal variation of the dimensionless fraction of the GIS to a freshwater flux. The details of the computation and the value of *α _GIS_* are given in Couplet *et al*. ^40^. Through this parameterization of *F _GIS_*, we establish a dynamic dependence of the AMOC on the state of the GIS that is useful for studying tipping cascade interactions between the AMOC and the GIS, as in Couplet *et al*. ^40^. Finally, a collapse of the AMOC will stabilize the melting of the GIS due to its regional cooling ^5^. In Equation (2) the second forcing variable *Ψ* is the state of the AMOC. By using a parameterization (1 – *Ψ*) this ensures that the stabilizing effect of the AMOC on the GIS is maximum when the AMOC has collapsed ( *Ψ* = 0).

The functions *μ _Ψ_* ( *Ψ*) and *μ _V_* ( *V*) are inverse time scales that describe the intrinsic dynamics of the tipping elements ^32^. Denoting either of the two state variables as *x*, these functions are defined as follows,

$$\;\mu_{x}(x)=\{\begin{array}{c} \begin{array}{ll} \frac{1}{\tau_{x}^{+}} & \;if\;\frac{dx}{dt}\;>\;0\;and\;0<x<1,\\ \frac{1}{\tau_{x}^{-}} & \;if\;\frac{dx}{dt}\;<\;0\;and\;0<x<1, \end{array}\\ 0\;if\;x\leq 0\;or\;x\geq 1. \end{array}\;(4)$$

We define two distinct time scales for the dynamics of the tipping elements to represent the asymmetry inherent in processes associated with the GIS, as ice melting typically occurs more rapidly than ice formation. $\tau_{x}^{+}$ represents the characteristic timescale of the intrinsic dynamics of the element transitioning from its collapsed state to its nominal state, whereas $\tau_{x}^{-}$ represents the characteristic timescale for the reverse transition, from the nominal state to the collapsed state. Here, we refer to the nominal state as the one with an active configuration of the AMOC. The third case in Equation (4) ensures that the normalized state of the tipping element, *x*, remains between the value *x* = 0 for the completely collapsed state and *x* = 1 indicating the initial pre-industrial state. The internal timescale dynamics are important for the evolution of tipping elements, and accurately estimating these parameters is challenging ^5, 29^. In the results section, we calibrate $\tau_{\Psi }^{+}$ and $\tau_{\Psi }^{-}$ based on a tuning procedure to reproduce the internal timescale dynamics of the AMOC in cGENIE. For the GIS, we set $\tau_{V}^{+}=5500$ years and $\tau_{V}^{-}=700$ years, based on reasonable values given in the reviews by Armstrong McKay *et al*. ^29^ and Couplet *et al*. ^40^.

### 2.2 Calibration module

Now, we aim to develop a calibration algorithm that, based on the critical coordinates of bifurcation points obtained from hysteresis experiments of complex models, will calibrate the parameters *a _i_,b _i_,c _i_,d _i_,e _ij_* ( *i,j* = 1,2, *i* ≠ *j*). We focus on validating the calibration methods for the AMOC. However, the translation to any simplified model based on the normal form of a bifurcation, such as Equations ( 1, 2), which include two forcings, will be reasonably straightforward. For instance, the translation for the GIS calibration is provided in the Supplementary Materials (see Data Availability). For the calibration, we seek to find the best fit for our double-fold structure to the complex hysteresis, ensuring that our simplified dynamics pass through the bifurcation points identified in the process-based hysteresis. In the paper describing the SURFER v2.0 model, Martinez-Montero *et al*. ^47^ develop a mathematical framework that allows, in the case of the Antarctic and Greenland ice sheet - also modelled by a canonical double-fold normal form - to be calibrated on experiments with more complex models by using the coordinates of the bifurcation points. From an epistemological standpoint, the significant advantage of the Martinez-Monteiro *et al*. ^47^ method is that it provides a clear link between the physically based tipping points coordinates and the calibration parameters. With this approach, the parameters are redefined such as to transparently describe the stability diagram. This method also allows to test the underlying hypothesis that the leading-order dynamics of tipping elements such as the AMOC can be captured by a double-fold. For these reasons, we aim to adhere to the calibration framework proposed by Martinez-Monteiro *et al*. ^47^. However, the mathematical framework presented by Martinez-Monteiro *et al*. ^47^ for modelling each individual ice sheet relies on a single ordinary differential equation with only one forcing parameter, namely the temperature anomaly. In our case, for the AMOC, we have two forcing parameters: the temperature anomaly and the freshwater flux.

To generalize the method of Martinez-Monteiro *et al*. ^47^ we need an operational assumption. Specifically, we assume that the complex models used to calibrate the hysteresis of the AMOC allow for independent application of forcing to the AMOC. In other words, if we take our ATCM model for the AMOC (see Equation (1)), we should be able to access process-based models that can force the AMOC solely through temperature anomaly while keeping the freshwater flux forcing constant, and vice versa. This is technically feasible with most models. If the aim is also to emulate the transition from the collapsed state to the nominal state, an additional constraint arises in the selection of complex models. Specifically, the complex models must be capable of conducting hysteresis experiments, which involve simulations where the tipping element remains in quasi-equilibrium. For instance, in the cGENIE experiments used for calibration of collapse dynamics, simulations span over 20,000 years (see Supplementary Materials in data availability). This requirement implies that models categorized as EMICs are generally suitable targets for emulation, as these models can perform simulations over extensive timescales while maintaining physical consistency ^36^. Throughout the text, we will refer to EMICs as the "complex" models we aim to emulate, although the emulator can be calibrated using any process-based model of the AMOC.

The recipe for our calibration, which is generalizable to *N* forcings, is as follows. To calibrate the coupling parameters associated with the *N* forcing variables, we perform *N* independent sensitivity experiments to independently calibrate the associated bifurcation diagrams by systematically reducing the calibration to a Martinez-Monteiro *et al*. ^47^-type model with only one forcing variable, as the other *N* – 1 variables become constants. For the AMOC, *EXPA* is defined as the calibration experiment of the AMOC intensity with respect to a temperature forcing, conducted using any complex model that satisfies the aforementioned conditions. From this experiment we can retrieve the coordinates of the bifurcation points denoted by,

$$\;({\text{Ψ}}^{+},T^{+}),({\text{Ψ}}^{-},T^{-}).\;(5)$$

In this context, *Ψ* ^±^ represents the normalized values of the AMOC intensity, where the system transitions from its nominal stable equilibrium state (Ψ ^+^) to its collapsed equilibrium state, and from the collapsed equilibrium state (Ψ ^–^) back to the nominal equilibrium state. The *T* ^±^ values denote the critical temperature anomaly forcing at which the two preceding bifurcations occur within the system. The Equation (1) in the *EXPA* experiment can be written as,

$$\;\frac{d\text{Ψ}}{dt}=\left(-{\text{Ψ}}^{3}+a_{1}{\text{Ψ}}^{2}+b_{1}\text{Ψ}+c_{1}+d_{1}T+e_{12}F_{GIS}^{A}\right)\mu_{\text{Ψ}}(\text{Ψ}),\;(6)$$

where $F_{GIS}^{A}$ represents the arbitrary constant value of the freshwater flux forcing applied during the simulation in the complex model. Consequently, $c_{1}+e_{12}F_{GIS}^{A}$ is a constant term in this experiment, effectively reducing the conceptual model to a single-forcing experiment. This simplification enables the application of the calibration technique proposed by Martinez Montero *et al*. ^47^ to determine the parameters.

$$\;a_{1}=\frac{3({\text{Ψ}}^{-}+{\text{Ψ}}^{+})}{2}\;(7)$$

$$\;b_{1}=-3{\text{Ψ}}^{-}{\text{Ψ}}^{+}\;(8)$$

$$\;c_{1}=\frac{T_{\text{Ψ}}^{+}{\text{Ψ}}^{{-}^{2}}({\text{Ψ}}^{-}-3{\text{Ψ}}^{+})-T_{\text{Ψ}}^{-}{\text{Ψ}}^{{+}^{2}}({\text{Ψ}}^{+}-3{\text{Ψ}}^{-})}{2(T_{\text{Ψ}}^{-}-T_{\text{Ψ}}^{+})}-e_{12}F_{GIS}^{A}\;(9)$$

$$\;d_{1}=-\frac{{\left({\text{Ψ}}^{+}-{\text{Ψ}}^{-}\right)}^{3}}{2(T_{\text{Ψ}}^{+}-T_{\text{Ψ}}^{-})}\;(10)$$

We define *EXPB* as the second calibration experiment examining the AMOC intensity response to freshwater forcing, using the same complex model. In this experiment, the freshwater flux parameterization in the complex model is implemented as a hosing tipping experiment. This freshwater flux could originate from Greenland Ice Sheet melt or precipitation-evaporation anomalies. From this experiment, we can extract the coordinates of the bifurcation points,

$$\;({\text{Ψ}}^{+},F_{GIS}^{+}),({\text{Ψ}}^{-},F_{GIS}^{-}).\;(11)$$

*F _GIS_* represents the critical values of the freshwater flux forcing at which the bifurcation of the AMOC occurs. By defining T ^B^ as the constant value of the temperature anomaly imposed during the complex model experiment, Equation (1) can be expressed as:

$$\;\frac{d\text{Ψ}}{dt}=(-{\text{Ψ}}^{3}+a_{1}{\text{Ψ}}^{2}+b_{1}\text{Ψ}+c_{1}+d_{1}T^{B}+e_{12}F_{GIS})\mu_{\text{Ψ}}(\text{Ψ}).\;(12)$$

Here, *c*_1_ + *d*_1_ *T ^B^* represents the constant term, and the method proposed by Martinez Monteiro *et al*. ^47^ enables the determination of two new values for the following parameters,

$$\;c_{1}=\frac{F_{GIS}^{+}{\text{Ψ}}^{{-}^{2}}({\text{Ψ}}^{-}-3{\text{Ψ}}^{+})-F_{GIS}^{-}{\text{Ψ}}^{{+}^{2}}({\text{Ψ}}^{+}-3{\text{Ψ}}^{-})}{2\left(F_{GIS}^{-}-F_{GIS}^{+}\right)}-d_{1}T^{B},\;(13)$$

$$\;e_{12}=\frac{{\left({\text{Ψ}}^{+}-{\text{Ψ}}^{-}\right)}^{3}}{2\left(F_{GIS}^{+}-F_{GIS}^{-}\right)},\;(14)$$

Thus, based on the five unknowns ( *a*_1_, *b*_1_, *c*_1_, *d*_1_, *e*_12_), independent calibration experiments allows us to apply the methodological framework of Martinez Monteiro *et al*. ^47^ to a single forcing variable. This results in six equations with an over-determination of the independent parameter *c*_1_. The application of this method to calibrate the GIS model, Equation (2), is provided in the Supplementary Materials (data availability). For the GIS case, it requires a complex model capable of simulating its volume projections with independent forcing possibilities for the AMOC and temperature.

With this methodological framework, it is also possible to generalize the approach to include more than two forcing parameters. For instance, as demonstrated in the Supplementary (Data availability), an additional freshwater flux forcing term was incorporated into Equation (1) to calibrate the impact of variations in the evaporation-precipitation balance over the Atlantic basin under future scenarios. Mathematically, the generalization to *N* forcing variables results in obtaining 4 *N* – 2( *N* – 1) equations with 4 + ( *N* – 1) knowns, leading to *N* – 1 over-determined equations. As will be shown in the next section with the emulation of cGENIE, these over-determinations reflect the fact that the coefficient *c*_1_ is the shared constant across all calibrations of different sensitivity experiments. It represents the component of the model that must adjust for each sensitivity experiment while remaining consistent across all of them. What value, then, should be assigned to *c*_1_? Sensitivity experiments have shown that defining $c_{1}=c_{1}^{A},$ where $c_{1}^{A}$ is determined from Equation (9), yields the most accurate results for emulating the evolution of the AMOC based on complex models. This is because temperature forcing is the primary driver of AMOC collapse ^15^, as demonstrated, for instance, in CMIP5 experiments ^13^. Consequently, we prioritize achieving the best possible calibration for temperature forcing while allowing for a greater margin of error in the calibration of freshwater flux forcing. Therefore, we adopt the value of c _1_ as determined by the temperature sensitivity calibration experiment.

The final parameter in the ATCM module that needs to be calibrated is the nominal internal dynamic time scale of the AMOC, denoted as $\tau_{\Psi }^{+}.$ Based on Armstrong McKay *et al*. ^29^ we assume that the internal dynamic time scale of the AMOC from its nominal stable state to its collapse stable state is the same, i.e., $\tau \equiv \tau_{\Psi }^{+}={\text{τ}}_{\Psi }^{-}$ . To calibrate this parameter, the proposed experimental setup involves running a simulation with the complex model to be emulated, using a stepwise parametrized forcing of the freshwater flux while maintaining a fixed temperature forcing. For instance, the simulation, with a forcing duration of 2000 years to reach equilibrium, can start with a 0 Sv forcing. It then increases the forcing by 0.05 Sv to slow down the AMOC, decreases the forcing by 0.05 Sv until the value reaches -0.05 Sv, and repeats this procedure several times. The goal is to obtain a time series of the AMOC intensity with several instances where the AMOC is being reduced. Based on these experimental data, a manual tuning of *τ* within ATCM allows for determining the best value to replicate the characteristic time scale of AMOC dynamics in the complex model.

## 3. Results

In this section, we calibrate the ATCM using cGENIE, through three calibration experiments labelled *EXPA*, *EXPB*, and *EXPC*. The first two experiments are used to calibrate the coefficients *a*_1_, *b*_1_, *c*_1_, *d*_1_, *e*_12_ while *EXPC* is employed to calibrate the parameter *τ*. Second, we validate the effectiveness of the ATCM by simulating an AMOC trajectory under a forcing configuration different from the calibration experiments and comparing the results with the original cGENIE simulation. Finally, we integrate the ATCM into SURFER to demonstrate the emulator's capability to rapidly simulate the evolution of the AMOC while sampling the forcing space under numerous realistic emission scenarios.

### 3.1 Calibration with cGENIE

To validate the emulator, we applied the ATCM to cGENIE ^49^, an EMIC ^37^ that has already demonstrated its ability to simulate AMOC hysteresis ^14, 50^. cGENIE includes an ocean circulation model (3D), a dynamic-thermodynamic sea ice model (2D) and an atmospheric energy moisture balance model (2D). The ocean model accounts for the horizontal and vertical transport of heat, salinity and biogeochemical tracers. The circulation is simulated through advection, convection, and mixing ^50, 51^. cGENIE has a very simple atmospheric model, which results in a minimal effect of net changes in the Precipitation-Evaporation (P-E) balance over the North Atlantic due to global warming. However, if the ATCM is applied to emulate complex models that account for variations in the P-E balance, these would be captured in the calibration through the temperature anomaly. Following the experimental framework outlined in the Methods section, we conducted two independent sensitivity calibration experiments on the AMOC intensity using cGENIE.

The first experiment, labelled *EXPA*, consist of a 20,000-year simulation with a prescribed *CO*_2_ forcing designed to generate a global atmospheric mean temperature anomaly. The forcing is parameterized as a linear function, increasing from 280 ppm to 2,800 ppm. Through the internal dynamics of cGENIE, this forcing is translated into a global mean surface air temperature anomaly, starting from *T* = 10° *C* for the initial thousands of years and reaching *T* = 10° *C* after 20,000 years (Figure S1- See underlying data). This setup enables us to cover the plausible range of the AMOC tipping point location in terms of temperature forcing ^5, 29^ while ensuring near-equilibrium conditions for the AMOC.

The second independent sensitivity calibration experiment, labelled *EXPA*, involves a 20,000-year hosing simulation with freshwater flux forcing linearly increasing from 0 *Sv* to 0.2 *Sv* (Figure S2- See underlying data). These values are chosen to align with the plausible range of AMOC tipping point locations related to freshwater forcing ^18, 27^ and to be sufficient to trigger an AMOC collapse in cGENIE. The total salinity of the oceans is maintained constant in the simulation by applying a compensatory freshwater flux over the Pacific. The freshwater hosing is applied between 20° *N* and 50° *N* across the full width of the Atlantic (Figure S3- See underlying data) to reproduce the hosing region by Rahmstorf *et al*. ^27^. The durations of the *EXPA* and *EXPB* simulations are chosen to ensure that the AMOC is forced sufficiently slowly, allowing it to remain in near a stable state throughout the calibration experiment.

**Figure 2.**
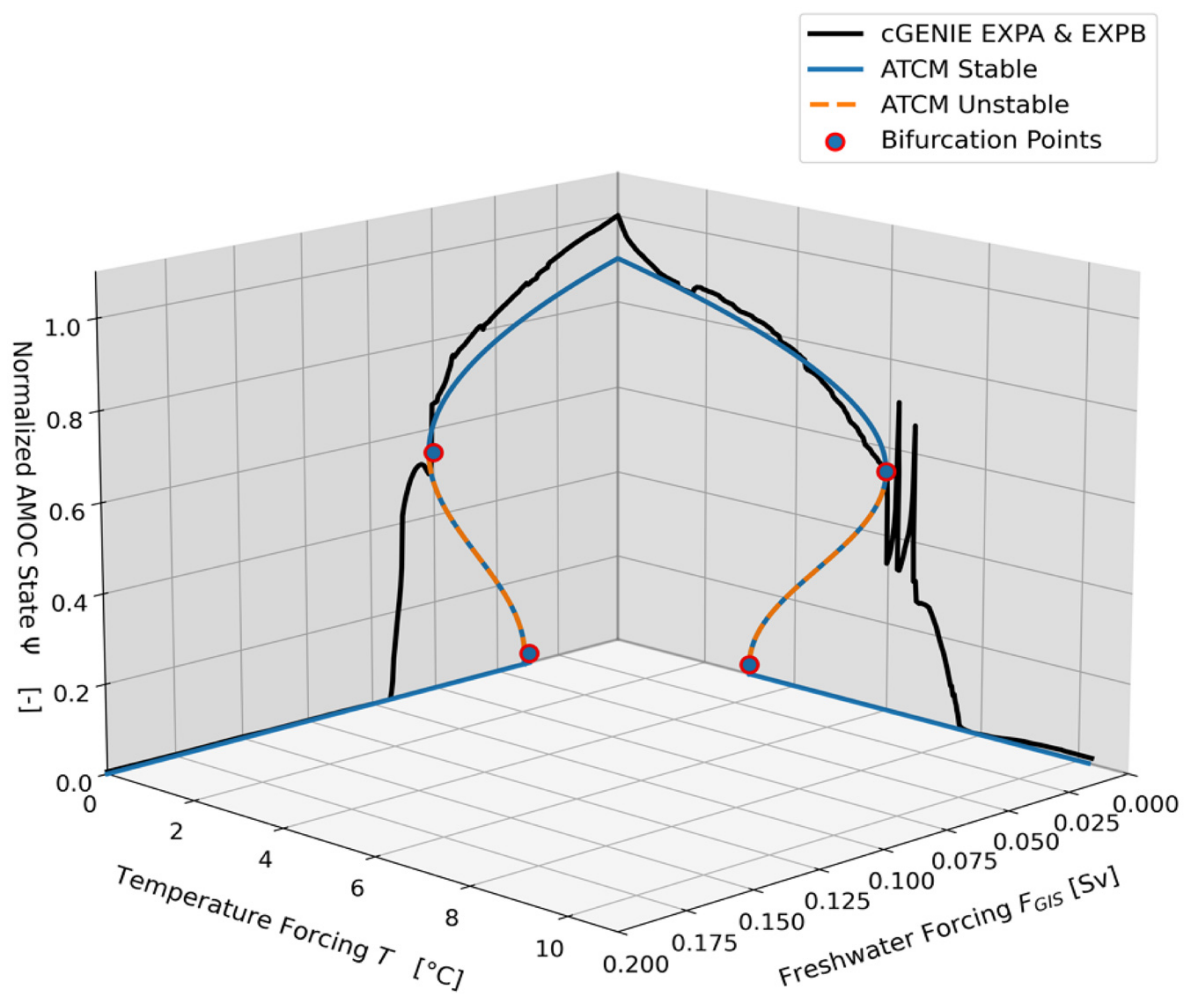
Bifurcation diagrams of cGENIE and ATCM in the ( *T, F _GIS_*) forcing space. The two simulations produced by cGENIE during the calibration experiments *EXPA* and *EXPB* are shown in their respective planes as solid black lines. The identification of the bifurcation points based on these collapsed branches is marked in red, while the simplified hysteresis produced by the ATCM is shown in blue. The branch of the unstable equilibrium, which separates the two stable equilibria, is represented by the orange dashed line.

The two trajectories of the AMOC intensity obtained with cGENIE in the *EXPA* and *EXPB* experiments are shown in Figure 2. Since the primary objective of the ATCM is to accurately calibrate the collapse branch, we focus exclusively on simulating collapse scenarios with cGENIE. This allows to extend the duration of the simulations with the complex model and thus, obtain an AMOC as close as possible to its equilibrium in the complex model. In other words, a complete hysteresis cycle was not performed to refine our calibration of the collapse branch, but this does not undermine the validity of the results.

As demonstrated in Laridon ^52^, the ATCM can also successfully emulate the AMOC trajectory of the complex model through the second bifurcation point. However, to achieve an optimal fit for the collapse trajectory, slight deviations from the bifurcation point coordinates provided by the complex model may improve the emulator's accuracy in representing the collapse trajectory. To calibrate the ATCM at the second bifurcation point (see Supplementary Materials in data availability), we therefore use the calibration simulation data from cGENIE provided in Laridon ^52^.

The coordinates of the bifurcation points are extracted from the experiments and are listed in Table 1. There is no established method for determining the coordinates of bifurcation points when analysing the hysteresis of a complex model. Consequently, the selection must rely on expert judgment and a thorough understanding of the complex model's behaviour. In the ATCM model, the points *Ψ* ^±^ for bifurcation must be the same, whether they are reached via temperature forcing or freshwater forcing. Moreover, we aim to find the best fit for temperature forcing, so we define $\Psi^{\pm }\equiv \Psi_{EXPA}^{\pm }.$ Using these values, we compute the parameters ( *a*_1_, *b*_1_, *c*_1_, *d*_1_, *e*_12_), according to Equation ( 7– 10, 14), and the results are presented in Table 2. Based on the calibration of these parameters, the simplified hysteresis loop emulated by the ATCM is shown in Figure 2. Since we calibrated the ATCM using the *c*_1_ value from *EXPA*, the double-fold structure correctly passes exactly through the two bifurcation points identified on the cGENIE hysteresis curve from the temperature forcing calibration experiment. However, this is not the case for *EXPB*, as will be explained in the Discussion section. Indeed, there is a 0.002 *Sv* difference between the $F_{GIS}^{+}$ tipping point identified from cGENIE and the value computed by the ATCM, resulting in a relative difference between the two of 2.53%. Overall, the collapse dynamics demonstrate a reasonably good calibration against the cGENIE simulations.

**Table 1.** Bifurcation coordinates. The values are retrieved from the cGENIE calibration sensitivity experiments *EXPA* and *EXPB* shown in Figure 2. The $F_{GIS}^{-}$ value is retrieved from cGENIE hysteresis (see Supplementary Materials in data availability). The *T* ^–^ value was adjusted to improve the calibration of the upper branch.

	Ψ ^+^ [ *Adim*]	Ψ ^–^ [ *Adim*]	T ^+^ [° *C*]	T ^–^ [° *C*]	$F_{\mathrm{GIS}}^{+}$ [ *Sv*]	$F_{\mathrm{GIS}}^{-}$ [ *Sv*]
***EXPA***	0.545	0.022	5.93	3	/	/
***EXPB***	0.545	0.022	/	/	0.075	0.037

**Table 2.** Internal dynamics parameter. The parameters are computed with the ATCM using Equation ( 7– 10, 14) using coordinates of the bifurcation points given by Table 1.

*a*_1_	*b*_1_	*c*_1_	*d*_1_	*e*_12_
0.851	-0.036	0.074	-0.024	-1.882

**Figure 3.**
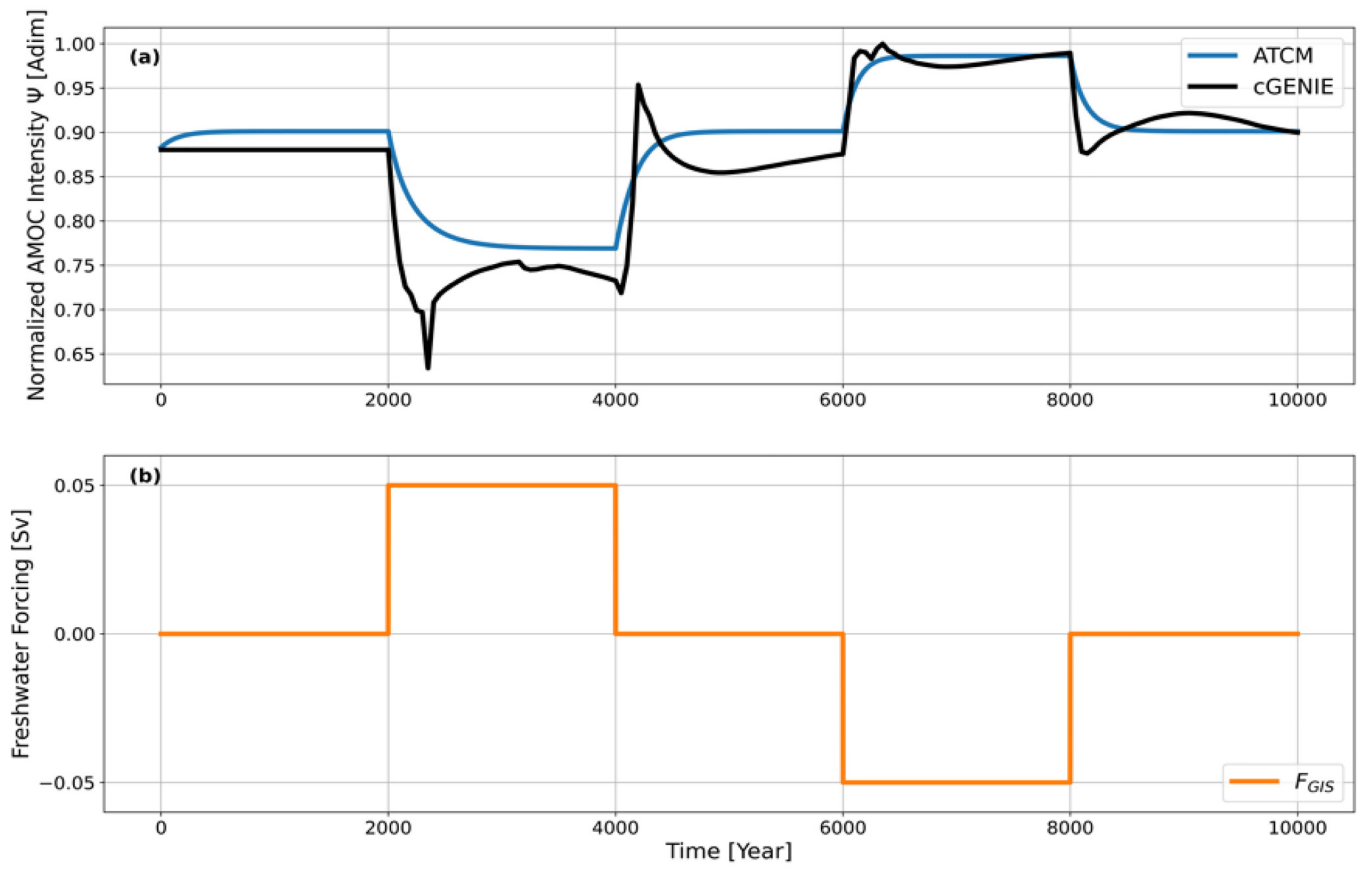
( **a**) Normalized AMOC intensity in the ATCM (blue) and cGENIE (black) under varying freshwater flux forcing. ( **b**) Parameterization of freshwater flux forcing in the calibration experiment *EXPC*.

The final parameter to calibrate in the ATCM to emulate cGENIE is the parameter *τ*, which represents the nominal timescale of the intrinsic dynamics of the AMOC. We apply the experimental protocol described in the Methods section, conducting a calibration experiment labelled *EXPC* with cGENIE. In this experiment, a step-by-step parameterized forcing of the freshwater flux is applied (see Figure 3b).

By doing so, the AMOC is modified to diagnose the time required to transition between equilibrium states under the applied forcing. In Figure 3a, the trajectory of the AMOC simulated by cGENIE is shown in black, while the trajectory simulated by the ATCM is shown in blue. The goal is to calibrate the parameter *τ* to reproduce the time lag between the intensity of the AMOC and its corresponding equilibrium intensity under the applied forcing. After calibration, a reasonable trajectory of the ATCM compared to cGENIE is achieved with a value of *τ* = 140 yr ^-1^.

Differences emerge at the various plateaus between the ATCM and cGENIE because the AMOC in cGENIE has not yet fully stabilized to its equilibrium state, and because more complex dynamics are incorporated in cGENIE. Additionally, the difference in the initial equilibrium state of the AMOC between cGENIE and the ATCM arises from discrepancies in the preindustrial AMOC intensity values in cGENIE and the ATCM (see Figure 2). These discrepancies will be discussed in the following section. Nevertheless, they do not compromise the calibration of the parameter *τ*, as it is designed to replicate the temporal lag between the AMOC intensity at a given moment and its corresponding equilibrium intensity during the four forcing level transitions in *EXPC*.

### 3.2 Validation of the emulator

To validate the emulator, we compare the ability of the ATCM to simulate the behaviour of the AMOC in cGENIE using a new experiment, referred to as *val_exp_*1. This experiment represents a combination of forcing conditions distinct from those used in the calibration experiments. The *val_exp_*1 simulation spans 20,000 years and combines the thermal forcing from *EXPA* — a linear increase in *CO*_2_ concentration from 280 ppm to 2,800 ppm (see Figure S1- See underlying data) — with a linear freshwater forcing increase from 0 *Sv* to 0.2 *Sv* (see Figures S2 and S3- underlying data).

**Figure 4.**
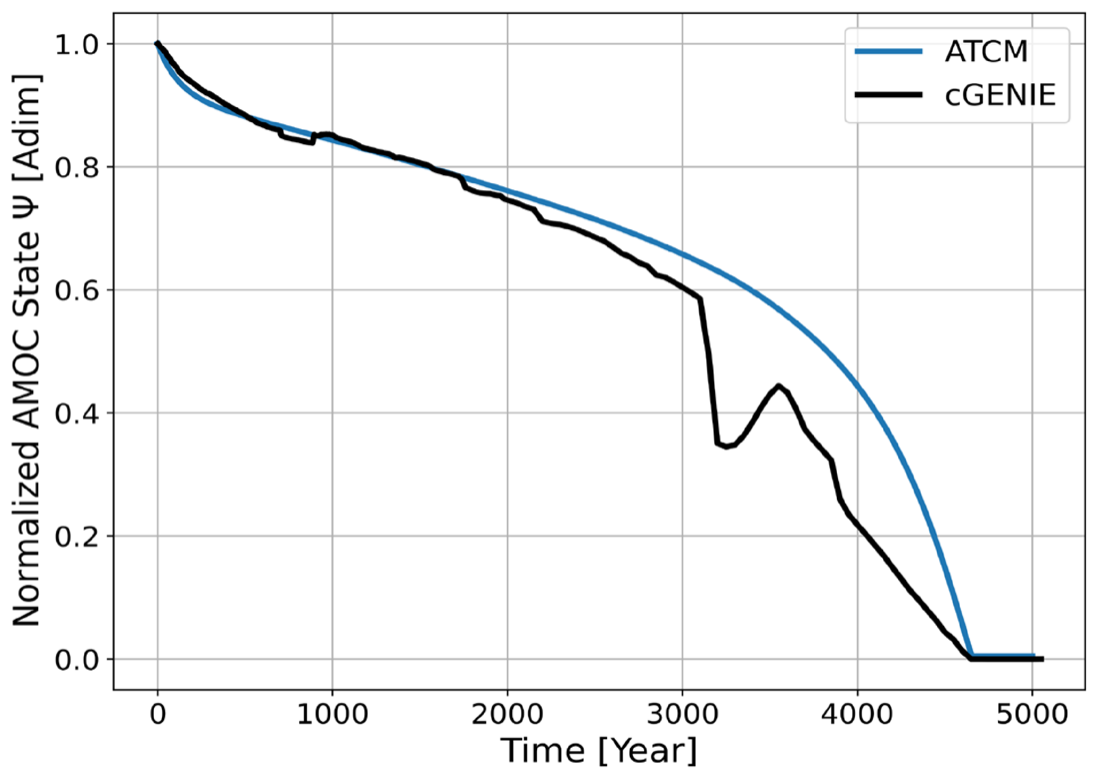
Trajectories of the AMOC in the ATCM emulator (blue) and cGENIE (black) in the *val_exp_*1 validation experiment with combined global mean temperature and Greenland Ice Sheet freshwater forcing.

The trajectories of the normalized AMOC intensity in both the emulator and the complex model are shown in Figure (4). The ATCM, despite its simplified dynamics, captures the trajectory described by cGENIE reasonably well, particularly during the first 3,000 years of the simulation. However, after 3,000 years, a sudden drop in AMOC intensity, followed by a partial recovery, is not captured by the ATCM. This discrepancy ultimately leads to a notable difference of 26% in the AMOC intensity after 3200 years. However, the parameters of the ATCM have been calibrated to replicate the moment of complete collapse in the AMOC intensity as given by the complex model, and we observe a difference of less than 5 years between cGENIE and the ATCM. As previously highlighted, the abrupt decrease in AMOC intensity in the ATCM during the early centuries of the simulation is due to differences in the equilibrium state associated with zero temperature forcing between the ATCM and cGENIE, which result from the calibration process.

### 3.3 ATCM integration within the SURFER climate model

**Figure 5.**
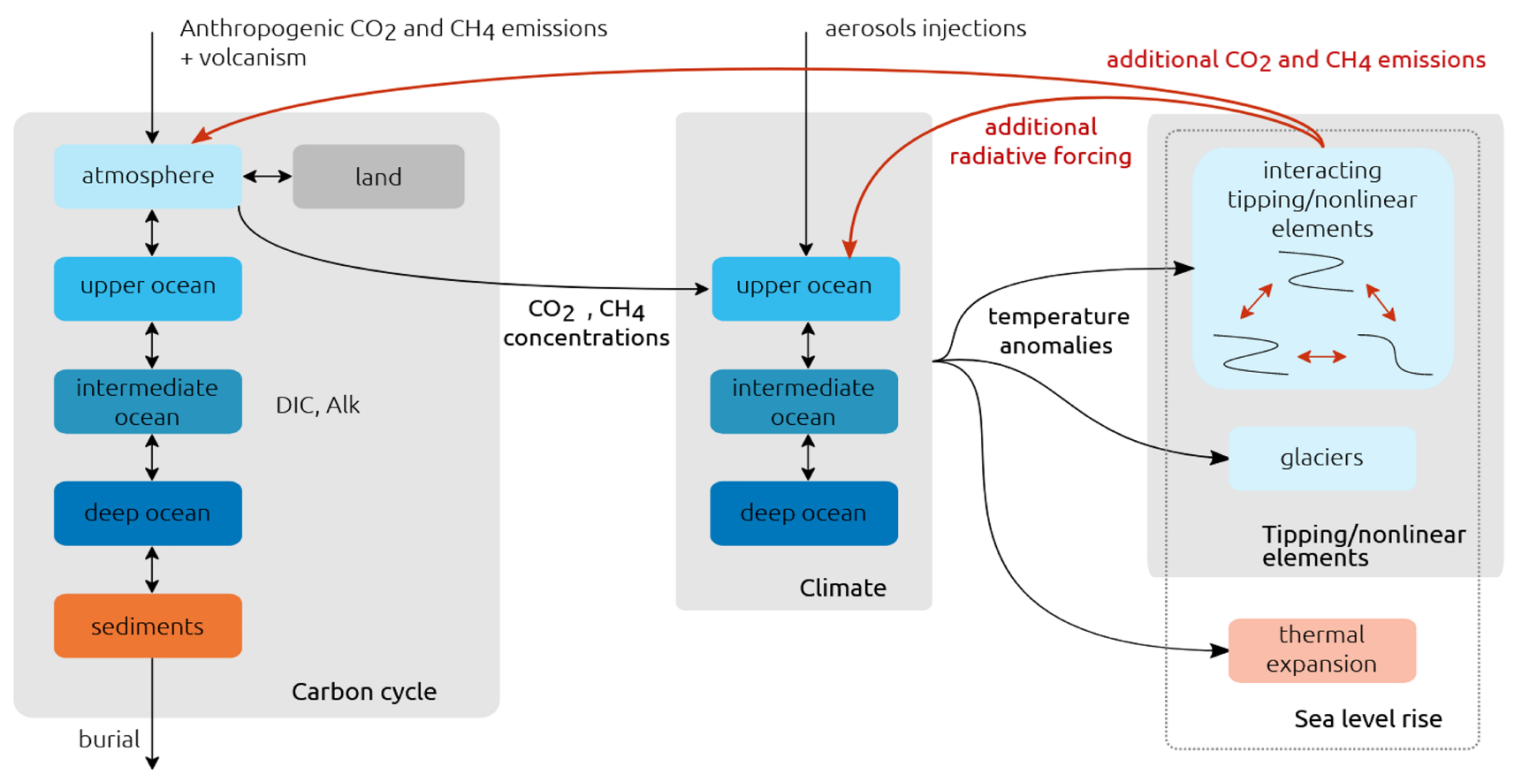
Conceptual diagram of SURFER, including interacting tipping elements and their feedback on climate. Figure reproduced with permission from Couplet *et al*. ^40^. The ATCM is integrated into the 'Tipping Elements' box of SURFER.

We now introduce the ATCM emulator into the reduced-complexity climate model SURFER (see Figure 5). SURFER features a process-based carbon cycle capable of reliably simulating atmospheric CO₂ concentrations and global mean temperature changes. This reduced-complexity model also simulates sea-level rise and various ocean acidification metrics in response to anthropogenic greenhouse gas emissions, while enabling simulations over timescales ranging from decades to millions of years ^47, 48^. Version 3.0 of SURFER comprises 17 differential equations that describe carbon exchanges among six reservoirs: the atmosphere, terrestrial systems, upper, intermediate, and deep ocean layers, and deep-sea sediments ^48^. Additionally, it models temperature anomalies across ocean layers, ice sheet volumes for Greenland and Antarctica, and sea-level changes due to glacier dynamics.

SURFER has proven effective for integrating tipping element dynamics into climate simulations, as by Couplet *et al*. ^40^. At the time of this project, version 3.0 of SURFER was not yet available. Therefore, we chose to integrate the ATCM into a preliminary version of v3.0 (see Data Availability) which we here refer to as pre3.0. There are no major differences between versions pre3.0 and 3.0; notably, the changes pertain to the parameterization of SURFER’s carbon cycle, which is not central to this study's objective of constructing an AMOC emulator coupled to a reduced-complexity climate model. To integrate the ATCM into SURFER pre3.0, we decided to deactivate all components associated with tipping elements other than the AMOC and GIS.

Incorporating the ATCM emulator into a reduced-complexity climate model like SURFER provides a robust tool for examining a broad phase space and a wide range of realistic emission scenarios. This approach maintains realistic parameterizations while ensuring computational efficiency and manageable runtime. Figure 5 illustrates the fifteen full-span emission scenarios selected as inputs to simulate the evolution of the AMOC within the emulator once integrated into SURFER. These 15 scenarios represent cumulative carbon emissions ranging from 1000 to 5000 PgC into the atmosphere from 1750 to 2500, covering the spectrum from SSP1-2.6 to SSP5-8.5. After 2500 AD, greenhouse gas emissions are set to zero for the remainder of the simulations in all scenarios.

### 3.4 Critical manifold and sampling the forcing space

**Figure 6.**
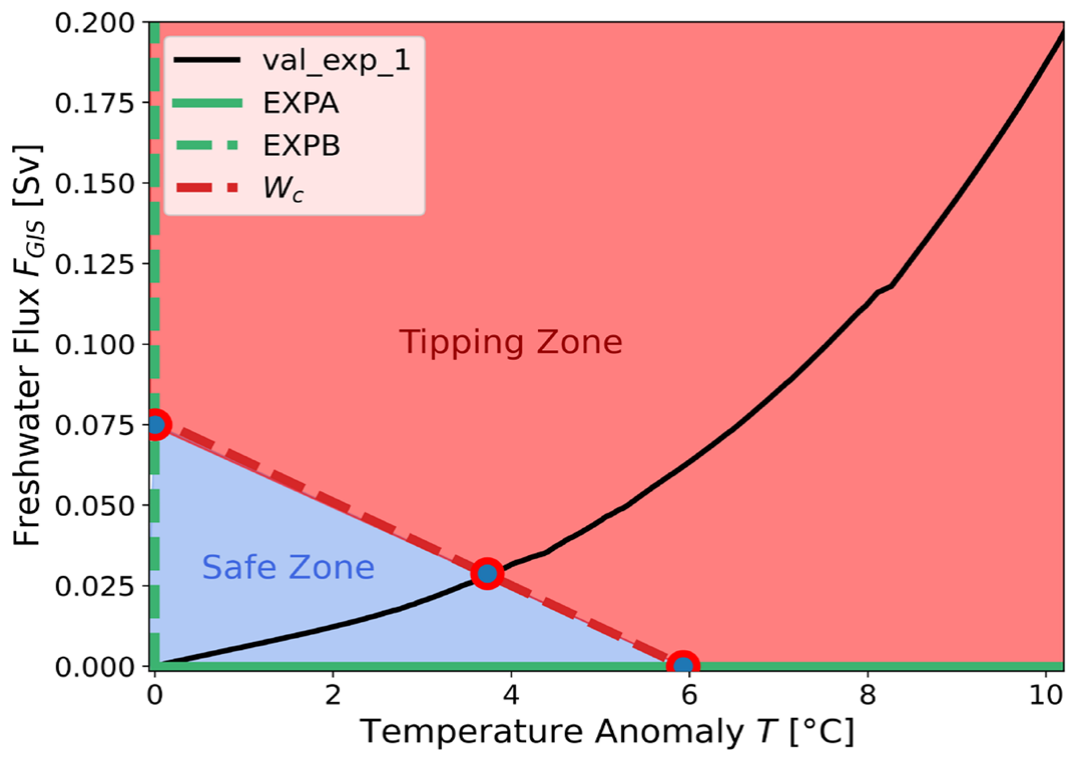
Representation of the different forcings applied in the two calibration experiments, *EXPA*, *EXPB* and the *val_exp_*1 validation experiment. The tipping points identified in the two calibration experiments are represented by the blue and red points. The red dashed line, *W _c_*, defines the critical bifurcation manifold in the ATCM. The “Safe Zone” in the forcing space corresponds to the set of linear combinations of the two forcing variables that do not reach the bifurcation point in the ATCM. The “Tipping Zone” exceeds this bifurcation point.

Based on the calibration of the ATCM to cGENIE, we can represent (see Figure 6) the two calibration experiments, *EXPA* and *EXPB*, as well as the validation experiment *val_exp_*1, in the forcing variable space ( *T*, *F _GIS_*). This representation demonstrates the use of the emulator as a tool for mapping the forcing space of a given complex model. The critical manifold *W _c_* is defined by the ATCM as follows,

$$\;W_{c}(T)=\frac{d_{1}}{e_{12}}(T^{+}-T).\;(15)$$

This manifold delineates the forcing space into two regions: one where the linear combinations of the variables *T* and *F _GIS_* do not lead to a tipping of the AMOC in the emulator, and another where such combinations would eventually cause the AMOC to tip into an alternative equilibrium state. Despite the approximations inherent in the calibration process, the ATCM provides a valuable pre-diagnostic tool for analysing forcing combinations that, if sustained for a sufficient duration ^31^, are expected to lead to a complete tipping of the AMOC.

**Figure 7.**
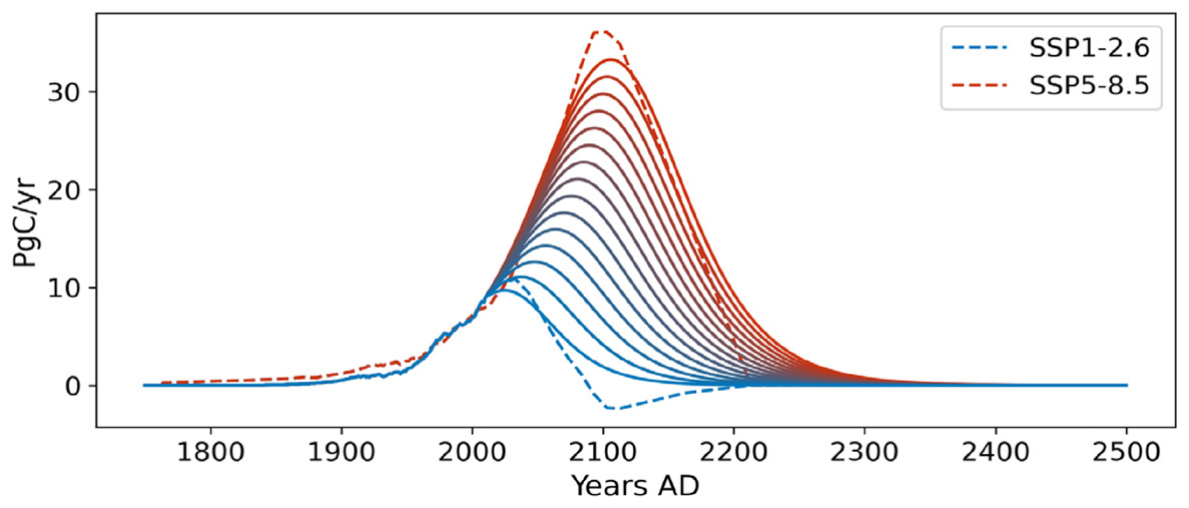
Fifteen full-span emission scenarios, covering the emission space from SSP1-2.6 to SSP5-8.5, are used in SURFER. These scenarios account for cumulative emissions from 1000 to 5000 PgC into the atmosphere since 1750. The emission trajectories range from blue for scenarios with the lowest emissions to red for scenarios with the highest greenhouse gas emissions.

**Figure 8.**
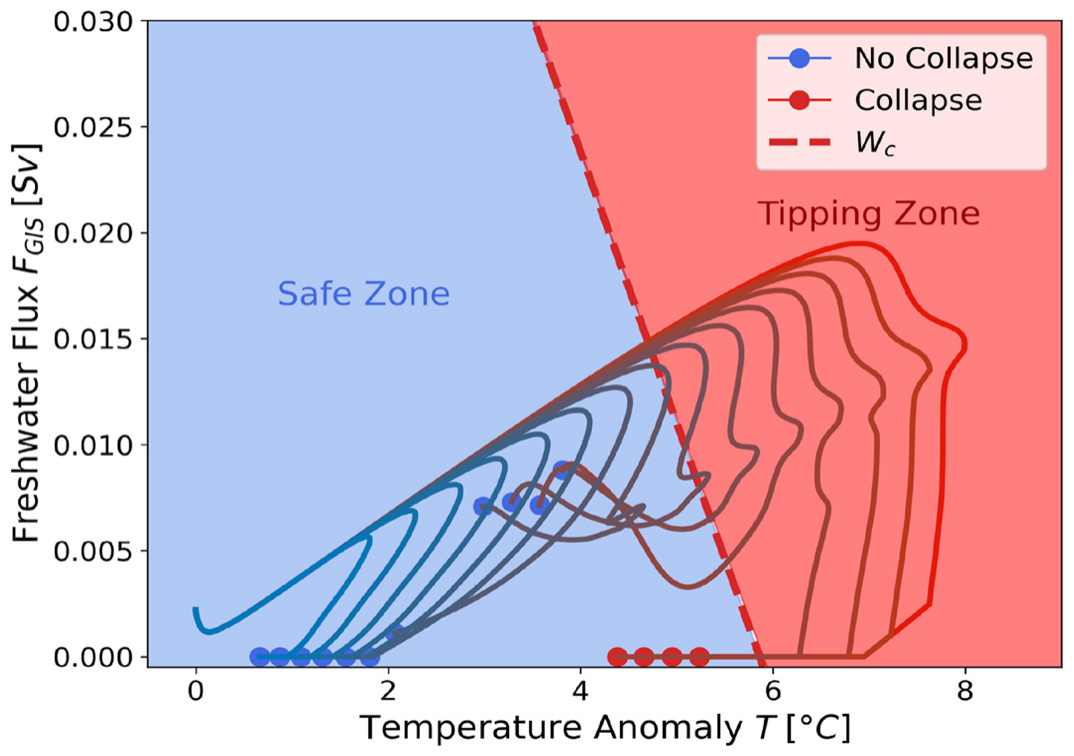
Representation of the 15 emission scenarios spanning SSP1-2.6 to SSP5-8.5 (see Figure S4- See underlying data) in the ( *T, F _GIS_*) forcing space. **Their associated temperature anomaly and freshwater flux contributions are given by SURFER.** The dots indicate the state of the AMOC at the end of the 10 000-year simulation. A green dot signifies *Ψ* > *Ψ* ^+^ corresponding to an active AMOC state, while a red dot indicates *Ψ* < 0.1, representing the collapsed state. The red dashed line, *W _c_*, denotes the critical bifurcation manifold in the ATCM. The “Safe Zone” in the forcing space corresponds to the set of linear combinations of the two forcing variables that remain below the bifurcation threshold identified in the ATCM. Conversely, the “Tipping Zone” represents conditions that exceed this threshold.

As an application, we studied the response of the ATCM once integrated into SURFER under 15 different emission scenarios spanning SSP1-2.6 to SSP5-8.5 (see Figure 7). Figure 8 illustrates the trajectories of these 10 000-year simulations in the forcing space ( *T*, *F _GIS_*). The critical manifold *W _c_* is the same as in Figure 5, obtained from the ATCM through the two calibration experiments *EXPA* and *EXPB* conducted with cGENIE. We observe that for 8 out of the 15 trajectories, the linear combination of the two forcing variables exceeds the bifurcation threshold of the AMOC. However, for four of these trajectories, the intensity and duration of this exceedance are sufficiently low to result in the overshoot-without-tipping phenomenon identified by Ritchie *et al*. ^31^. Moreover, hysteresis phenomena are observed, as the four simulations with the highest greenhouse gas emissions that cause the collapse of the AMOC fail to restore its circulation, even when forcing levels return below critical thresholds. Finally, Figure 6 and Figure 8 highlight the importance of considering two forcing variables in the case of the AMOC, as the inclusion of freshwater flux as a forcing variable significantly lowers the temperature-associated tipping point due to their combined effects.

### 3.5 New collapse trajectories captured by the emulator

**Figure 9.**
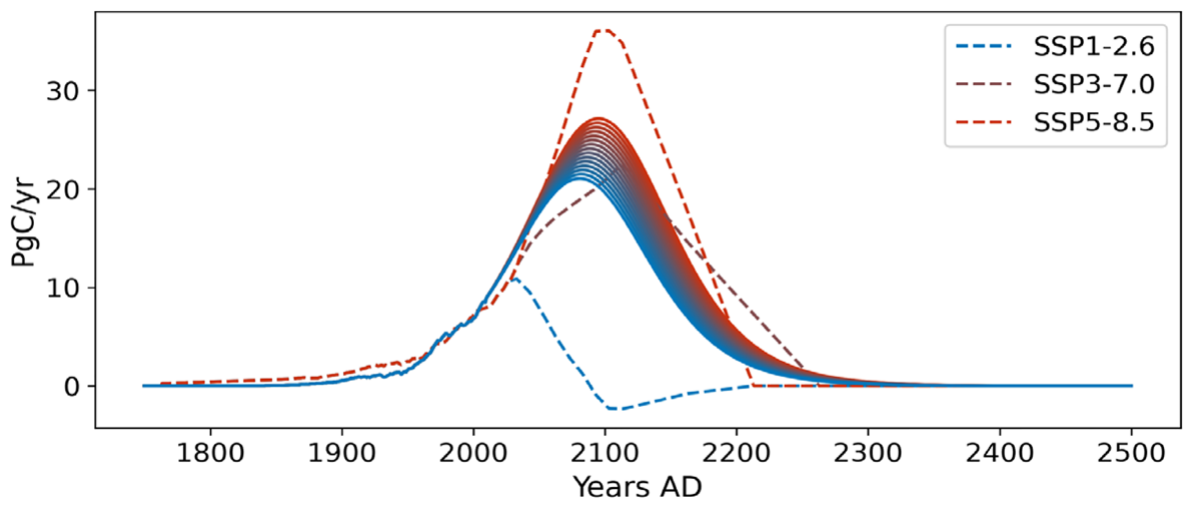
Fifteen mid-range emission scenarios, spanning SSP3-7.0 to SSP5-8.5, are used in SURFER to study the interaction of temperature and freshwater flux forcings. These scenarios account for cumulative emissions from 3000 to 4000 PgC into the atmosphere since 1750. The emission trajectories range from light blue for scenarios with the lowest emissions to dark red for scenarios with the highest greenhouse gas emissions.

To demonstrate the importance of developing an AMOC emulator that accounts for both temperature and freshwater flux forcing associated with GIS melting, we compared the AMOC evolution predicted by the ATCM in SURFER with the case where the freshwater flux forcing is intentionally disabled. 15 greenhouse gas emission scenarios are used, spanning total emissions from 3000 PgC to 4000 PgC. This places their emission peaks between those of the SSP3-7.0 and SSP5-8.5 scenarios ( Figure 9).

**Figure 10.**
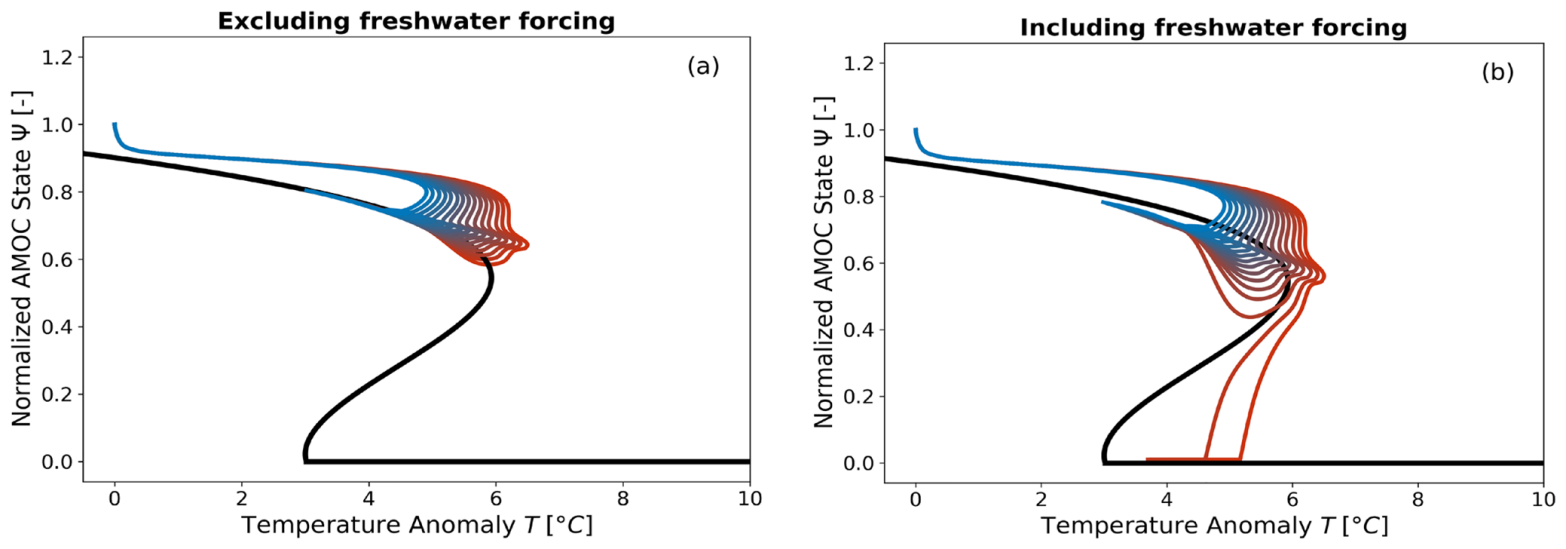
Trajectories of the AMOC in the temperature bifurcation diagram with the freshwater flux forcing deactivated Figure 10 **a** and activated Figure 10 **b**. The simulations run for 10,000 years. The cGENIE-calibrated double-fold dynamics of the equilibrium are represented by the black line. The trajectories simulated by SURFER are shown in colors corresponding to total emission scenarios ranging from 3000 PgC to 4000 PgC (see Figure 9).

In the case where the forcing by freshwater flux from GIS melting is deactivated (see Figure 10a), the 15 trajectories do not lead to an AMOC collapse during these 10,000-year simulations. However, overshoot without tipping phenomena are observed for the three highest emission scenarios, as the AMOC is subjected to a global mean temperature anomaly exceeding its tipping point identified at *T* ^+^ = 5.93 °C. When the forcing with *F _GIS_* is activated and calibrated with cGENIE, as developed in this paper, we observe (see Figure 10b) that for the two highest emission scenarios (3928.57 PgC and 4000 PgC), the AMOC collapses. One new additional overshoot without tipping trajectory (3642.86 PgC) is also observed. Thus, new dynamics with substantial impacts have been captured through the development of an emulator that accounts for both freshwater flux forcing from GIS melting and temperature forcing.

## 4. Discussion

The objective was to develop an AMOC emulator based on double-fold dynamics incorporating two forcing variables—globally averaged temperature and freshwater flux— that could be effectively calibrated using complex models. To achieve this, we generalized the calibration method of Martinez Monteiro *et al*. ^47^ by assuming the feasibility of conducting independent forcing experiments for calibration.

The resulting AMOC Tipping Calibration Module (ATCM) has proven highly effective, as demonstrated in the Results section and Figure 2. It successfully adjusts its simplified double-fold structure to closely replicate the cGENIE complex model simulations. In the calibration based on cGENIE experiments, the differences between the tipping points associated with *F _GIS_* obtained from cGENIE and those simulated by the ATCM are minimal. Specifically, this difference of 0.002 *Sv*, equivalent to a 2.5% error, falls within a reasonable uncertainty range reported in the literature ^26, 28^. To calibrate the characteristic timescale parameter *τ*, an effective experimental design with the complex model was demonstrated.

The differences between the ATCM and cGENIE data in the calibration experiments can be attributed to two primary factors: the inherent limitations of the conceptual approach and constraints within the ATCM calibration module. The first is the inherent limitations of the simplified approach in fitting a double-fold structure to a complex hysteresis. These first limitations were known a priori. From a technical perspective, fitting a third-order polynomial to a complex curve is expected to result in a potentially significant discrepancy between the approximation and the reality described by the complex model. The challenge, therefore, lies in identifying the most effective method to minimize this inherent error as much as possible. Here, we explain the origins of these approximations and the measures taken to mitigate them.

Technically, the first source of error in the calibration module arises from the values of the independent term *c*_1_, which must take the same value across all sensitivity experiments. As outlined in the Methods section, the coefficient is over-determined across the two sensitivity experiments. Consequently, a specific value of *c*_1_ is selected based on one calibration experiment, but this value may not apply to the other. This approach implies that our simplified model is precisely calibrated to the bifurcation points of one calibration experiment (temperature forcing) but not the other (freshwater flux forcing). However, the decision to minimize error for temperature forcing is justified because this forcing is the dominant factor influencing the AMOC ^13, 14, 53^.

A second limitation of the ATCM calibration module is its inability to accurately align with the pre-industrial value of the AMOC. In their single-forcing parameter model, Martinez Montero *et al*. ^47^ were able to mathematically constrain the calibration model such that, under zero temperature anomaly forcing, the initial state of the simplified model corresponded precisely to the pre-industrial value of the tipping element. However, this approach cannot be generalized to models with two forcing parameters, as implementing such a constraint would introduce additional restrictions on the constant forcing values $F_{GIS}^{A}$ and *T _B_*, thereby limiting experimental flexibility. However, although the difference between the actual equilibrium state of AMOC intensity during the pre-industrial period and that reproduced by the ATCM is significant (10%), it does not substantially affect the emulator's relevance. This is because, under forcings such as those associated with climate change, the system initially evolves out of equilibrium. Moreover, since the nominal timescale parameter τ has been calibrated based on cGENIE, the ATCM effectively replicates the initial trajectory of the AMOC under forcing, as shown in Figure 4.

Once the simplified dynamics of the AMOC were calibrated, the emulator was tested on a new experiment combining both forcing variables. The emulator successfully replicated the dynamics of cGENIE up to the point where physical processes not accounted for by the ATCM caused significant deviations in the AMOC's collapse dynamics relative to cGENIE. Consequently, these simplifications in the emulator's dynamics led to a notable discrepancy of 26% in the intensity of the AMOC at a specific point in its collapse trajectory. However, the primary value of constructing tipping element emulators, such as the present case for the AMOC, lies in their ability to assess, at low computational cost and with fidelity to more complex models, the predicted timing of the tipping element's total collapse. In this regard, the comparison between the ATCM and the *val_exp_*1 experiment from cGENIE demonstrates that only a difference of a few years separates the prediction of the complex model from that of the emulator. Over a 20,000-year simulation, this highlights the robustness of the ATCM.

Therefore, we argue that the resulting emulator, which utilizes independent forcing experiments from complex models to calibrate tipping elements characterized by a double-fold bifurcation and two forcing variables, is relevant. The calibration produced provides a tool that is sufficiently close to the reality of the complex model being emulated to be usable as an emulator. However, complex models, whether EMICs or even ESMs, still carry significant uncertainty regarding the exact location of the AMOC tipping points and, consequently, the timing of their collapse ^5, 29, 53^. In this context, the ATCM should not be regarded as a tool for delivering precise quantitative projections. While it is highly likely that the model values deviate from exact accuracy, we demonstrate that they are sufficiently well calibrated to remain plausible. This is the key requirement for an AMOC tipping element emulator, enabling improved studies of the AMOC state under likely positions in its forcing space. As demonstrated in the Results section (see Figure 7), the ATCM facilitates realistic sampling of the forcing space once integrated into a reduced-complexity climate model like SURFER. Where complex models face significant computational constraints, the presented emulator enables a large number of simulations to be performed at a much lower cost while maintaining consistency with more complex models. These computationally-efficient simulations also allow for the study of overshoot without tipping scenarios ^31^ by sampling the bivariate forcing space with higher resolution ( Figure 8).

Moreover, the emulator allows assessing, under the assumption that the two forcings add linearly, the critical manifold *W _c_* of the emulated complex model. Comparing these critical varieties provides a method for assessing the inherent sensitivities of each model to AMOC collapse. A promising direction for future research would be to calibrate the ATCM using an ensemble of EMICs and possibly ESMs, allowing for the exploration of both the forcing space and the structural model uncertainty space. Furthermore, it has been demonstrated that incorporating freshwater flux forcing from GIS melting into the AMOC emulator is crucial, as it allows for the capture of two new collapse and one overshoot without tipping scenarios that could not previously be modelled with temperature forcing alone. The two emission scenarios that now lead to AMOC collapse in the emulator have emission peaks falling between those of the reference scenarios SSP3-7.0 and SSP5-8.5. This underscores the urgent need to reduce greenhouse gas emissions to prevent triggering tipping points such as AMOC collapse, in addition to mitigating baseline global warming.

Lastly, the calibration method presented here can be further generalized to include additional forcing variables (see Supplementary in data availability). Another promising avenue is applying the ATCM methodology to the Greenland Ice Sheet (GIS) (see Supplementary Materials in data availability), creating a non-linear dynamics tool calibrated on complex models to explore possible cascading collapses between the AMOC and GIS ^32, 34^. Beyond the GIS, this approach to constructing tipping element emulators can be extended to other tipping elements, beginning with the six additional elements whose initial parameterizations are included in SURFER v3.0 ^40^. Moreover, this emulator could be integrated into other reduced-complexity climate models, such as FAIR ^54^, with minimal technical difficulty, as it requires only the global mean temperature anomaly and a parameterization of the GIS freshwater flux as input variables from the climate model.

## 5. Conclusion

We have presented the AMOC Tipping Calibration Module (ATCM), an emulator of the AMOC tipping element dynamics based on a double-fold structure that, for the first time, incorporates two forcing variables that can be calibrated using experiments from complex models. To address this challenge, we adopted the methodological hypothesis that independent calibration experiments for each forcing variable could be conducted using the complex system. It was demonstrated that the constructed emulator satisfactorily reproduces the AMOC behaviour observed in the target complex model. Of course, the emulator is constrained by its simplified dynamics, which introduce noticeable differences between its outputs and those of the emulated complex model, cGENIE. Nevertheless, the ATCM is capable of reproducing the timing of the total AMOC collapse predicted by the complex model with an error of less than five years. This result falls within the uncertainty range reported in the literature for complex models, ensuring that the emulator’s outputs remain both plausible and consistent with those of the complex model. Furthermore, by accounting for both globally averaged temperature anomalies and freshwater fluxes associated with Greenland Ice Sheet (GIS) melting, the emulator effectively incorporates this key dynamic in AMOC collapse. It has thus been demonstrated that the emulator can capture two new AMOC collapse trajectories and one overshoot trajectory without tipping for emission scenarios with peaks between those of SSP3-7.0 and SSP5-8.5. These trajectories could not be reproduced using temperature forcing alone. In addition, the ATCM offers significant computational advantages, characterized by much shorter runtimes compared to complex models. When integrated into a reduced-complexity climate model such as SURFER, the ATCM enables rapid yet realistic exploration of the forcing space under various emission scenarios. This emulator also facilitates a retrieval technique for extracting the critical manifold of the complex model within the forcing space. It provides a valuable framework for investigating boundary cases of overshoot without tipping in complex models and for comparing their sensitivities to AMOC collapse, thereby enabling the exploration of structural model uncertainty.

Finally, the calibration methodology presented here can be readily extended to other tipping elements. A promising next step would be to calibrate a GIS emulator in a similar manner, creating a tool capable of modelling cascading collapses between the AMOC and GIS when calibrated on a complex model. The methodology behind the emulator could also be applied to other tipping elements within SURFER or other reduced-complexity climate models. Therefore, beyond the tool itself, this study has developed and validated a calibration method for simplified two-forcing tipping element dynamics based on complex models, such as EMICs and ESMs. This work establishes a framework for bridging the gap between conceptual and complex models, advancing our understanding of future tipping element dynamics. It also underscores the urgent need for immediate and robust mitigation policies to address the risks associated with the potential collapse of these critical systems.

## Ethics and consent

Ethics and consent were not required.

## Data Availability

Laridon A *et al*. 2025. Supplementary Materials. Connecting complex and simplified models of tipping elements: a nonlinear two-forcing emulator for the Atlantic meridional overturning circulation. Zenodo. DOI: https://doi.org/10.5281/zenodo.14979157 ^55^ This repository contains the supplementary materials referred in this paper (i) GIS calibration (ii) parameterization of the AMOC with three forcing variables (iii) more details on the cGENIE experiments used for calibration (iv) cGENIE hysteresis used for calibration of the second tipping point. License: Creative Commons Zero v1.0 Universal.

## Software availability

Laridon, A *et al*. 2025. Connecting complex and simplified models of tipping elements: a nonlinear two-forcing emulator for the Atlantic meridional overturning circulation. Zenodo. DOI: https://doi.org/10.5281/zenodo.14944240

This repository contains the data as well as the scripts, the SURFER model and the ATCM used in this study. The *ATCM.ipynb* Jupyter Notebook contains the Python code where the ATCM is defined, calibrated, and validated using cGENIE simulations. The *SURFER_pre3.0_ATCM.ipynb* Jupyter Notebook contains the integration of the ATCM into the SURFER pre3.0 climate model. These two notebooks were used to generate all the figures presented in this paper. The emission scenario data required to run the simulations implemented in SURFER can be found in the *data/SURFER/* directory within the

License: Creative Commons Zero v1.0 Universal.

The code for the version of the ‘muffin’ release of the cGENIE Earth system model used in this paper, is tagged as v0.9.61, and is assigned a DOI: https://doi.org/10.5281/zenodo.15021902. Configuration files for the specific experiments presented in the paper can be found in the directory: genie-userconfigs/PUBS/published/Laridon_ *et_al*_2025. Details of the experiments, plus the command line needed to run each one, are given in the readme.txt file in that directory. All other configuration files and boundary conditions are provided as part of the code release.

License: MIT

A manual detailing code installation, basic model configuration, tutorials covering various aspects of model configuration, experimental design, and output, plus the processing of results, is assigned a DOI: https://doi.org/10.5281/zenodo.13377225.

License: CC-BY 4.0
