# Supplementary material for: Connecting complex and simplified models of tipping elements: a nonlinear two-forcing emulator for the Atlantic meridional overturning circulation
Source: Open Res Eur. 2025 Mar 31;5:87. [Version 1] doi: 10.12688/openreseurope.19479.1 (PMC12612620; doi:10.12688/openreseurope.19479.1)
Supplement: Peer review history

# Reviewer report: Reviewer response for version 1

**DOI:** 10.21956/openreseurope.21075.r54425

**Published:** 2025-06-18

**Authors:** Dipti Hingmire

**Affiliations:** 1 University of Victoria, Victoria, British Columbia, Canada

Complex systems like AMOC require computationally expensive complex Earth System models to be run to understand its tipping points. Here the authors have developed a nonlinear two forcing emulator for AMOC and it calibrated with reduced complexity climate models.  New insights regarding AMOC collapse from SSP3-7.0 to SSP5-8.5 are provided with calibrated emulator. 

 More and more emulators of this kind should be developed and calibrated for rapid and less expensive way of exploring tipping points.  Therefore, this study is important and relevant.

Is the study design appropriate and does the work have academic merit?

Yes

Is the work clearly and accurately presented and does it cite the current literature?

Yes

If applicable, is the statistical analysis and its interpretation appropriate?

Yes

Are all the source data underlying the results available to ensure full reproducibility?

Yes

Are the conclusions drawn adequately supported by the results?

Yes

Are sufficient details of methods and analysis provided to allow replication by others?

Yes

Reviewer Expertise:

I am expert of large scale climate dynamics including AMOC dynamics, however I lack mathematical expertise to evaluate emulator design and concepts. I request editor please provide extra weightage to other reviewers comment before making decision.

I confirm that I have read this submission and believe that I have an appropriate level of expertise to confirm that it is of an acceptable scientific standard.

# Reviewer report: Reviewer response for version 1

**DOI:** 10.21956/openreseurope.21075.r54758

**Published:** 2025-06-16

**Authors:** Valerian Jacques-Dumas

**Affiliations:** 1 Utrecht University, Utrecht, The Netherlands

**Short summary**

 This article presents a simple tipping elements and tipping cascades emulator, which can be tuned to the dynamics of more complex models. The authors model the tipping behaviour of the AMOC and GIS as coupled double-fold bifurcations and tune their parameters to independent hysteresis experiments in the cGENIE model. The methodology for tuning these parameters already existed but was limited to a single forcing variable. The authors extended it to an arbitrary number of forcing variables and show that they are able to reasonably reproduce the dynamics of cGENIE.

 This simple emulator is then coupled to SURFER, where it is used to explore the response of the AMOC to a large range of SSP scenarios. This approach allows the derivation of the critical manifold in the forcing space; the inclusion of multiple forcings also shows new overshoot and tipping trajectories that could not be observed before. 

**Review summary: Approved with reservations**

 Tackling the issue of computational cost is very important to study tipping cascades and, more generally, tipping elements. Using simple emulators tuned to complex dynamics is an appealing way to solve this problem so I find the overall method, the results and perspectives of this study to be interesting and worth publishing. However, I have some comments and general remarks that should be addressed to enhance readability and reproducibility.  

**Major comments**

 1) My major concern is the readability of the article. Many sentences are confusing and I had to read them multiple times before understanding. Moreover, some grammatical mistakes also hinder readability. Some examples are given below but I would suggest to read again through the article carefully.

-          (Section 1.3) “However, the AMOC is influenced by two forcing variables: temperature anomaly and freshwater flux. Given the necessity of more realistically constraining potential future AMOC trajectories, this study seeks to address the following objective: develop an AMOC emulator with two forcing parameters — temperature and freshwater flux — that can be calibrated against hysteresis from complex models and integrated into a climate model.” 

        This is redundant and overly complicated. “However” at the beginning is also wrongly used, leading to some confusion.

-         (Section 2.2) “Mathematically, the generalization to N forcing variables results in obtaining 4N – 2(N – 1) equations with 4 + (N – 1) knowns, leading to N – 1 over-determined equations. As will be shown in the next section with the emulation of cGENIE, these over-determinations reflect the fact that the coefficient c1 is the shared constant across all calibrations of different sensitivity experiments. It represents the component of the model that must adjust for each sensitivity experiment while remaining consistent across all of them.” 

        This could be made simpler and clearer.

-         (Section 2.2) “For instance, the simulation, with a forcing duration of 2000 years to reach equilibrium, can start with a 0 Sv forcing. It then increases the forcing by 0.05 Sv to slow down the AMOC, decreases the forcing by 0.05 Sv until the value reaches -0.05 Sv, and repeats this procedure several times. The goal is to obtain a time series of the AMOC intensity with several instances where the AMOC is being reduced.”

        This is VERY confusing. It took me several attempts to understand it and I thought it would be a linear ramping-up so Figure 3b came as a surprise. It could be rephrased more simply and more efficiently.

-         (Section 3.1) “Nevertheless, they do not compromise the calibration of the parameter τ, as it is designed to replicate the temporal lag between the AMOC intensity at a given moment and its corresponding equilibrium intensity during the four forcing level transitions in EXPC.” 

        The end of this sentence is unclear.

-         (Section 3.1)”The goal is to calibrate the parameter τ to reproduce the time lag between the intensity of the AMOC and its corresponding equilibrium intensity under the applied forcing.” 

        This sentence is unclear, partly because of the expression “intensity of the AMOC”

-         (Section 4) “While it is highly likely that the model values deviate from exact accuracy, we demonstrate that they are sufficiently well calibrated to remain plausible.”

        This sentence is very unclear, especially in relation with the previous sentence. What do you mean here? Could you rephrase it?

 2) Across the article, authors mention that hysteresis are performed on a single forcing parameter while keeping the other fixed at certain value T^B or F_GIS^A. However, what precise values are given other than these variables are never explicit in the Results section. This must be important, though for the calibration of the ATCM. Can you make it clearer? Figure 2 suggests that these extra parameters are fixed to 0 but it should be in the text. After re-reading, I understood that the fixed values are the ones presented in Table 1, is it correct? If so, it should be made clearer in the text.

 3) Moreover, Figure 2 is not very clear. You could consider making it 2 panels, one for each variable. 

 4) Similarly, I would strongly encourage you to replace Figure S4 with two panels because the bifurcation points in the intertwined planes are not readable at all. In particular, I cannot check from the Figure that the values of T- and F_GIS you mention are the right ones.

 5) The other important point is the generalisation of your results. You claim that their emulator is very flexible in reproducing tipping behaviour, which is very appealing, but validate it using the same parameters as for calibration. As far as I understand, the experiment val_exp_1 simply combines the parameter ramp-ups of EXPA and EXPB. It would be very interesting to see how the emulator performs when validated (against cGENIE) for different hosing experiments and global warming scenarios. This would also support the interesting dynamics observed when the emulator is applied to a whole range of SSP scenarios. 

 6) In the Supplementary Material S1, you go through the whole calibration procedure for the GIS but do not present any result. I find it a pity that you show the whole method to potentially simulate AMOC-GIS tipping interaction, but stop there. Could you consider adding these results to the paper (if there is enough room)? Otherwise, is it worth keeping this Section of the Supplementary Material? If the AMOC-GIS interaction results are out of the scope of this paper, I think that the calibration of the GIS model is also out of its scope. 

 7) In relation with this point, at the very end of Section 2.1, you write “In the results section, we calibrate τ + Ψ and τ − Ψ based on a tuning procedure to reproduce the internal timescale dynamics of the AMOC in cGENIE. For the GIS, we set V+ τ = 5500 years and V− τ = 700 years, based on reasonable values given in the reviews by Armstrong McKay et al.[29] and Couplet et al.[40].” 

- Why mention the time scales of the GIS if you do not use them at all afterwards? And if you use them later, you should rather give at the same place where you explain the calibration of AMOC time scales (end if Section 2.2).

7) (Section 3.1) “Since the primary objective of the ATCM is to accurately calibrate the collapse branch, we focus exclusively on simulating collapse scenarios with cGENIE.” 

- You describe the whole procedure to calibrate all parameters of your emulator (including the return time scale tau_minus) and even provide in Supplementary Material (Section S4) the full hysteresis experiments which would allow you to perform the whole calibration. Why not use it? Wouldn’t it make your results more robust by bringing additional constraint?

8) (Section 4) “Moreover, the emulator allows assessing, under the assumption that the two forcings add linearly, the critical manifold Wc of the emulated complex model.” 

- Could you discuss this assumption? Is it a strong assumption? Has it already been discussed elsewhere?

9) (Conclusion) Most of your Conclusion is redundant with your Discussion. Can you consider removing the conclusion and slightly expanding the discussion to avoid repeating the same things twice? 

 10) (Supplementary Material, Section 1). I don’t understand this section. Is it even possible in cGENIE to increase the temperature while keeping the AMOC strength constant? It seems very strange since you also claim that the P-E anomaly stemming from global warming is the main driver of AMOC weakening. Moreover, you never mention what is the fixed value of the AMOC strength. Similarly, when you vary the AMOC strength (how? Is it a model parameter?), you fix the global mean temperature but don’t explicitly give the value of T_V^D. You can leave most details in the MSc thesis you cite but everything needed for reproduction of your results should be in the article.

 11) In relation with this comment, the sentence “For the GIS case, it requires a complex model capable of simulating its volume projections with independent forcing possibilities for the AMOC and temperature.” in Section 2.2 is not very clear.

 12)

- (Section 3.1) “However, to achieve an optimal fit for the collapse trajectory, slight deviations from the bifurcation point coordinates provided by the complex model may improve the emulator’s accuracy in representing the collapse trajectory.”
- (Caption of Table 1) “The T– value was adjusted to improve the calibration of the upper branch.”
- (Supplementary Material, Section 4) “Additionally, to improve the calibration of the emulator on the collapse branch, it was shown in Laridon (52) that arbitrarily changing the coordinates of the second bifurcation point improves calibration. For this reason, in this study, we reuse the critical values of the second bifurcation point from these two experiments to focus on longer calibration runs that more effectively capture the AMOC collapse at the first bifurcation point, given that forcing can act more slowly in the calibration experiments described in the main text.” 

What does it mean that you “adjust” or “arbitrarily change” the value of T- to improve calibration? Moreover, I do not really understand the sentences quoted here from the Supplementary Material: they are very heavy and not clear at all.

**Minor comments**

 1) (Section 1.2) “However, due to their prohibitive computational demands, complex models, such as Earth System Models (ESMs) are unable to simulate numerous complete AMOC hysteresis curves[36].” 

- You already cite van Westen et. al (2024) but not here unfortunately. It would be particularly relevant because they precisely compute a hysteresis curve from an ESM.

2) (Section 2.1) “In Equation (1), this term encapsulates the physical effects of warming on AMOC water stratification, as well as freshwater anomalies resulting from simulated P-E in the complex model.”

- This is confusing because it seems that T represents at the same time temperature and salinity. Moreover, after that, you no longer mention its P-E component. You could thus simplify this sentence and leave the whole P-E flux to the Supplementary Materials.

3) (Section 2.2) “Based on Armstrong McKay et al.29 we assume that the internal dynamic time scale of the AMOC from its nominal stable state to its collapse stable state is the same, i.e., . Ψ ττ − +Ψ ≡ = τ”

- Has this actually been checked in sensitivity experiments?

4) (Section 3.1, paragraph 3) Don’t you mean EXPB instead of EXPA?

 5) (Section3.1, paragraph 3) “The freshwater hosing is applied between 20°N and 50°N across the full width of the Atlantic (Figure S3- See underlying data) to reproduce the hosing region by Rahmstorf et al.[27].”

- Could you comment on the influence of this choice? Different hosing areas have since then been compared, for instance in [1].

6) (Section 3.2) “However, after 3,000 years, a sudden drop in AMOC intensity, followed by a partial recovery, is not captured by the ATCM.”

- Do you know why such drop happens? Had it already been observed?

7) (Section 3.4) “As an application, we studied the response of the ATCM once integrated into SURFER under 15 different emission scenarios spanning SSP1-2.6 to SSP5-8.5.”

- Do you have any references on the design of these scenarios?

8) At the end of Section 3.3 and in Section 3.4, you reference Figure 5 twice, although it should be Figure 6.

 9) (Supplementary Material, Section 1)“We denote ��������, in the case of the GIS as the first calibration experiment of the GIS volume in relation to temperature.”

- This sentence should be rephrased for clarity.

10) (Supplementary Material, section 2) “The duration of �������� was choose to”

- The verb should be “chosen”. 

11) (Supplementary Material, Section 4) “For ��������, it was also a 20,000-year run,”

- This is not consistent with the beginning with the rest of the paragraph. From what I understand from the end of the paragraph, the full EXPB run lasts 20,000 years. But the EXPA run lasts 40,000 years. So in the sentence quoted here, you cannot use “also” and “20,000 years” together, otherwise it’s very confusing. In this same sentence, “-0.2 Sv” is cut, with “-“ on one line and “0.2 Sv” on the next, although they should stay together for clarity.

Is the study design appropriate and does the work have academic merit?

Yes

Is the work clearly and accurately presented and does it cite the current literature?

Partly

If applicable, is the statistical analysis and its interpretation appropriate?

Not applicable

Are all the source data underlying the results available to ensure full reproducibility?

Yes

Are the conclusions drawn adequately supported by the results?

Partly

Are sufficient details of methods and analysis provided to allow replication by others?

Yes

Reviewer Expertise:

Extreme and rare events, AMOC, nonlinear dynamics, machine learning

I confirm that I have read this submission and believe that I have an appropriate level of expertise to confirm that it is of an acceptable scientific standard, however I have significant reservations, as outlined above.

## References

[1] : Understanding AMOC stability: the North Atlantic Hosing Model Intercomparison Project. *Geoscientific Model Development* .2023;16(7) : 10.5194/gmd-16-1975-2023 1975-1995 10.5194/gmd-16-1975-2023

# Response

**Published:** 2025-10-17

**Authors:** Amaury Laridon

**Affiliations:** Water and Climate, Vrije Universiteit Brussel, Brussels, Brussels, Belgium

**Major comments**  

**1) Readability of the article** *“My major concern is the readability of the article. Many sentences are confusing and I had to read them multiple times before understanding. Moreover, some grammatical mistakes also hinder readability. Some examples are given below but I would suggest to read again through the article carefully.”* Thank you for raising this concern. We have carefully reviewed the entire manuscript and reformulated numerous sentences to enhance clarity and readability. Special attention was devoted to all highlighted sections, which have been systematically revised.  

**2) Clear mention of the value of the constant forcing parameter** *“Across the article, authors mention that hysteresis are performed on a single forcing parameter while keeping the other fixed at certain value T^B or F_GIS^A. However, what precise values are given other than these variables are never explicit in the Results section. This must be important, though for the calibration of the ATCM. Can you make it clearer? Figure 2 suggests that these extra parameters are fixed to 0 but it should be in the text. After re-reading, I understood that the fixed values are the ones presented in Table 1, is it correct? If so, it should be made clearer in the text.”* Indeed, it was an oversight that these fixed values were not specified in the *Results* section nor discussed further in the *Discussion*. We have now addressed this by explicitly stating the values chosen for** *F* *GIS* *A* and *T* *B* in the *Results* section and by including them in Table 1. In the *Discussion* section, we have added a paragraph presenting the scientific rationale behind our choice of setting** *F* *GIS* *A* *=0 Sv* and *T* *B* *=0 °C*, as well as the implications of using alternative fixed but non-zero values for these two parameters.  

**3) Figure 2 not very clear** *“Figure 2 is not very clear. You could consider making it 2 panels, one for each variable.”* We appreciate the reviewer’s suggestion. However, we would prefer to keep Figure 2 in its current form, as we believe that presenting the full forcing space in a single panel effectively conveys the range of possible combinations between temperature and freshwater flux. This unified representation is intended to emphasize that only two calibration experiments are sufficient to approximate the complex system dynamics within the entire forcing space. The specific values of the coordinates corresponding to the bifurcation points identified in the figure are provided in Table 1 for clarity. Following the reviewer’s comment, we have added a sentence in the manuscript, when introducing this figure, to further highlight its purpose of providing a visual representation of the now two-dimensional forcing space. This addition further supports our argument that the emulator will subsequently enable sampling across this wide parameter space. Finally, we have included in the Supplementary Materials the two 2D plots of the bifurcation diagrams for temperature and freshwater forcing alone, in order to enhance readability.  

**4) Figure S4 not very clear and values used for** ***T*** ***-*** **and** ***F*** ***GIS*** ***-*** **​** *Similarly, I would strongly encourage you to replace Figure S4 with two panels because the bifurcation points in the intertwined planes are not readable at all. In particular, I cannot check from the Figure that the values of T- and F_GIS you mention are the right ones.* Thank you for raising this concern. We have ultimately decided to remove Section 4 from the Supplementary Materials. The main reason is that this section contained an error and did not contribute to the clarity of the presentation; on the contrary, it was confusing. Specifically, the values chosen for *T*and *F* *GIS* *-*​ are not directly those from the simulation results presented in A. Laridon’s MSc thesis. As previously stated—though perhaps not clearly enough—these values were artificially modified in the emulator, based on the rationale that the focus of this study is to demonstrate that a good calibration on the collapse branch, which is of greater near-term interest, can be achieved. It is also now reminded in Section 3.1 that there is a trade-off in calibrating the emulator to minimize the calibration error where it is most relevant given the scientific objective. Section 4 was originally included to show that it is also possible to calibrate the emulator on a full hysteresis curve, but its presentation and the way it was referenced in Section 3.1 were confusing. We have therefore rewritten the explanation of the choices and justifications for the values of *T* *-*and *F* *GIS* *-*​ ​, while inviting readers interested in the full hysteresis calibration results to consult the MSc thesis.  

**5) Generalization of the results** *The other important point is the generalisation of your results. You claim that their emulator is very flexible in reproducing tipping behaviour, which is very appealing, but validate it using the same parameters as for calibration. As far as I understand, the experiment val_exp_1 simply combines the parameter ramp-ups of EXPA and EXPB. It would be very interesting to see how the emulator performs when validated (against cGENIE) for different hosing experiments and global warming scenarios. This would also support the interesting dynamics observed when the emulator is applied to a whole range of SSP scenarios.* We thank the reviewer for this very relevant and constructive suggestion. In response, we now validate the emulator against four new scenarios in the revised manuscript: SSP2-4.5 and SSP5-8.5, each with and without an added hosing flux of 0.06 Sv. We provide a more detailed description of the experimental protocol in the updated manuscript. In addition, we performed a new calibration experiment for the timescale-related parameter, which allows a strict separation between the experiments used for calibration and those used for validation. We believe that these additions further reinforce the conclusions already presented in the previous version of the study.  

**6) Method developed for AMOC-GIS tipping interaction but not used** *In the Supplementary Material S1, you go through the whole calibration procedure for the GIS but do not present any result. I find it a pity that you show the whole method to potentially simulate AMOC-GIS tipping interaction, but stop there. Could you consider adding these results to the paper (if there is enough room)? Otherwise, is it worth keeping this Section of the Supplementary Material? If the AMOC-GIS interaction results are out of the scope of this paper, I think that the calibration of the GIS model is also out of its scope.* We thank the reviewer for this thoughtful comment. In the revised manuscript, we have modified Figure 1 by removing the GIS equation, as it unnecessarily complicated the interpretation of the figure. This change also makes the parameter τ more explicit. In addition, we have moved the former Equation (2), which described the evolution of the GIS, from the Methods section to Subsection 3.3 of the Results, where we integrate the emulator into SURFER. Finally, we have removed the previous parameterization of the AMOC to GIS interaction, as it was partly arbitrary and based on the MSc thesis of A. Laridon. We now adopt exactly the same equation and calibration as in SURFER v2.0 (Martinez Monteiro et al., (2022)), ensuring greater consistency, transparency, and reproducibility in our results. The primary objective of this study was to demonstrate the feasibility of constructing a two-variable forcing emulator capable of reproducing consistent results when compared with a target complex model. We have included Section S1 in the Supplementary Materials to illustrate how the proposed mathematical framework can also be applied to the construction and calibration of a GIS emulator, thereby enabling the exploration of potential tipping cascades between the AMOC and the GIS. While some example applications of this methodology are presented in the MSc thesis of A. Laridon, we have not yet calibrated the simplified GIS model against a complex GIS model. The purpose of Section S1 is therefore to outline the theoretical and methodological groundwork for such an extension, rather than to present calibrated results. Given the focus of the present paper, a full calibration of the GIS model and a detailed analysis of potential AMOC–GIS interactions are beyond its scope. However, we agree that these represent promising avenues for future research. We have clarified this motivation and limitation in the revised manuscript to better reflect the role of Section S1, in addition to the modifications described above.  

**7) Choice of the** ***τ*** ***V*** ***+/-*** *In relation with this point, at the very end of Section 2.1, you write “In the results section, we calibrate τ + Ψ and τ − Ψ based on a tuning procedure to reproduce the internal timescale dynamics of the AMOC in cGENIE. For the GIS, we set V+ τ = 5500 years and V− τ = 700 years, based on reasonable values given in the reviews by Armstrong McKay et al.[29] and Couplet et al.[40].” Why mention the time scales of the GIS if you do not use them at all afterwards? And if you use them later, you should rather give at the same place where you explain the calibration of AMOC time scales (end if Section 2.2).* We thank the reviewer for pointing out this lack of clarity. We have now added the missing information regarding the values of τ *V* *+* et τ *V* *-* in section 3.3. As mentioned in the previous comment, we now use exactly the same equation to describe the dynamics of the GIS, as well as the calibration parameters, as in SURFER v2.0 (cfr. Martinez Monteiro et al.(2022)). In that paper, the GIS equation was calibrated against the results of a complex GIS model. By adopting exactly the same GIS equation here, we are also able to compare the emulator’s results—with and without freshwater flux forcing—more rigorously than in the first version of the manuscript.  

**8) Assumption that the two forcings add linearly** *(Section 4) “Moreover, the emulator allows assessing, under the assumption that the two forcings add linearly, the critical manifold Wc of the emulated complex model.” Could you discuss this assumption? Is it a strong assumption? Has it already been discussed elsewhere?* Assuming that the forcings combine linearly is a common methodological choice, largely because it offers a straightforward and tractable starting point. While there is no definitive evidence that this assumption strictly holds, the fact that few tipping experiments in complex models consider multiple forcings makes it challenging to adopt an alternative approach. The aim of the emulator is therefore to provide a tool that, under the most common and simplified assumption of treating the two dominant AMOC forcings linearly, enables an assessment of AMOC sensitivity across different complex models. As discussed further in the revised manuscript, adopting alternative values for *F* *GIS* *A* and *T* *B*​ would make it possible to test the robustness of the assumption that the two forcings add linearly. However, if future literature supports a new prevailing hypothesis regarding the analytical form of the combination of these two forcings, it would be entirely possible to reformulate the AMOC equation in the emulator and apply the same calibration method to it.  

**9) Repetitions between the Conclusions and the Discussions** *(Conclusion) Most of your Conclusion is redundant with your Discussion. Can you consider removing the conclusion and slightly expanding the discussion to avoid repeating the same things twice?* We acknowledge the reviewer’s comment regarding the perceived redundancy between the conclusion and the discussion sections. In response, we have expanded the discussion to include additional points, making it more comprehensive and revised the conclusion.  

**10) Supplementary Materials, Section 1 – More clear information for reproduction** *I don’t understand this section. Is it even possible in cGENIE to increase the temperature while keeping the AMOC strength constant? It seems very strange since you also claim that the P-E anomaly stemming from global warming is the main driver of AMOC weakening. Moreover, you never mention what is the fixed value of the AMOC strength. Similarly, when you vary the AMOC strength (how? Is it a model parameter?), you fix the global mean temperature but don’t explicitly give the value of T_V^D. You can leave most details in the MSc thesis you cite but everything needed for reproduction of your results should be in the article.* The purpose of Section 1 of the Supplementary Materials is purely to provide a technical demonstration of how the same calibration method can be applied to a simplified model of the GIS in order to construct an emulator. We have added a paragraph at the beginning of this section to clarify its objective and to emphasize that applying this framework requires the availability of a complex model of the GIS. cGENIE, in itself, is not capable of producing the two calibration experiments required to emulate the GIS, assuming its two main forcings are global mean atmospheric temperature anomaly and AMOC intensity. Indeed, cGENIE does not include an explicit representation of the GIS or its melt processes. In this section, we describe in the protocol of *EXPC* that, using a suitable complex GIS model, a first sensitivity experiment should be conducted in which the GIS is forced by the atmospheric temperature anomaly while keeping AMOC intensity fixed. Using the notation of the section, the value is denoted as Ψ *V* *=*Ψ *V* *C*​. Similarly, for the second calibration experiment *EXPD*, the atmospheric temperature anomaly is held constant at a generic value *T* *V* *=* *T* *V* *D*​. It is expected that these numerical values are not provided, as these simulations have not been conducted. Once again, the sole purpose of this section is to present the mathematical framework and to highlight the operational constraints that such an application would impose on the complex GIS model to be used. We have also revised the citation of the MSc Thesis to make it clearer to the reader what can be expected from it. In the MSc Thesis, the GIS model was not calibrated based on a complex GIS model either; instead, parameter values were chosen based on reasonable assumptions drawn from the literature (e.g., Armstrong McKay et al. (2022)). However, the work did investigate the coupled emulator framework between the AMOC and GIS, and the potential tipping dynamics of both systems.  

**11) Section 2.2 – Need of a complex model for the GIS** *In relation with this comment, the sentence “For the GIS case, it requires a complex model capable of simulating its volume projections with independent forcing possibilities for the AMOC and temperature.” in Section 2.2 is not very clear.* As explained in our response to comment number 6, we have now relocated and revised the presentation of the GIS dynamics used in the manuscript. It is now strictly based on that of SURFER v2.0, which has already been published and was calibrated against a complex GIS model.  

**12) Arbitrarily change the value of T-** *(Section 3.1) “However, to achieve an optimal fit for the collapse trajectory, slight deviations from the bifurcation point coordinates provided by the complex model may improve the emulator’s accuracy in representing the collapse trajectory.”  ○  (Caption of Table 1) “The T– value was adjusted to improve the calibration of the upper branch.”  ○  (Supplementary Material, Section 4) “Additionally, to improve the calibration of the emulator on the collapse branch, it was shown in Laridon (52) that arbitrarily changing the coordinates of the second bifurcation point improves calibration. For this reason, in this study, we reuse the critical values of the second bifurcation point from these two experiments to focus on longer calibration runs that more effectively capture the AMOC collapse at the first bifurcation point, given that forcing can act more slowly in the calibration experiments described in the main text.”  ○  What does it mean that you “adjust” or “arbitrarily change” the value of T- to improve calibration? Moreover, I do not really understand the sentences quoted here from the Supplementary Material: they are very heavy and not clear at all.* As previously stated, we have decided to remove Section 4 of the Supplementary Materials, as it did not contribute to improving the clarity of the manuscript. As mentioned in response to Point (4) above, we have also revised Section 3.1 to clarify which bifurcation point coordinates are derived from the two cGENIE sensitivity experiments that are shown, and which coordinates are manually fixed to improve the calibration of the upper collapse branch. We now provide a clearer justification for this choice in Section 3.1. Accordingly, for the parameters Ψ *-* *,* *F* *GIS* *-* *,* *T* *-*, we have also updated the description of Table 1 to make it explicit that these values are manually set.  

**Minor comments**  

**1) Nuance on van Westen et al.(2024) citation** *(Section 1.2) “However, due to their prohibitive computational demands, complex models, such as Earth System Models (ESMs) are unable to simulate numerous complete AMOC hysteresis curves[36].” You already cite van Westen et. al (2024) but not here unfortunately. It would be particularly relevant because they precisely compute a hysteresis curve from an ESM.* We have added a reference to the study by van Westen et al. (2024) in this part of Section 1.2, emphasising that although hysteresis curves from ESMs do now exist, they remain scarce due to the high computational cost associated with producing them.  

**2) P-E mention in the T-forcing** *(Section 2.1) “In Equation (1), this term encapsulates the physical effects of warming on AMOC water stratification, as well as freshwater anomalies resulting from simulated P-E in the complex model.” This is confusing because it seems that T represents at the same time temperature and salinity. Moreover, after that, you no longer mention its P-E component. You could thus simplify this sentence and leave the whole P-E flux to the Supplementary Materials.* This has been addressed by removing the statement in Section 2.1 suggesting that the variable T accounts not only for the effect of heat on stratification but also for freshwater anomalies related to precipitation-minus-evaporation (P–E). That statement was indeed confusing. However, the explanation that the effect of P–E can be captured if it is also simulated by the complex model has been retained in Section 3.1. In this order, we believe the reading is much clearer.  

**3) Verification of tau_+ = tau_- in sensitivity experiments** *(Section 2.2) “Based on Armstrong McKay et al.29 we assume that the internal dynamic time scale of the AMOC from its nominal stable state to its collapse stable state is the same, i.e., .* *Ψ* *ττ* *− +* *Ψ* *≡ =* *τ* *”  ○ Has this actually been checked in sensitivity experiments?* These two parameters by themselves do not represent the total time required for the AMOC to tip from its ON state to its OFF state and vice versa, but rather characterize the internal dynamic time scales associated with the AMOC’s strengthening and weakening phases. Based on the analysis presented in Gérard and Crucifix (2024)1, Figures 7a and 7b illustrate hysteresis loops of AMOC intensity in response to atmospheric temperature anomalies and freshwater flux forcing. From these figures, we observe that, to a first approximation, the system spends a comparable amount of time transitioning from the ON to the OFF state as it does from the OFF to the ON state. Specifically, in both cases, the transition appears to take approximately 2000 years, which supports our assumption that *τ* *Ψ* *+* *=*τΨ *-*.  

 1 Gérard J, Crucifix M. Diagnosing the causes of AMOC slowdown in a coupled model: a cautionary tale. Earth System Dynamics. 2024 Mar 22;15(2):293–306.  

**4) Typo** *(Section 3.1, paragraph 3) Don’t you mean EXPB instead of EXPA?* Yes, indeed — thank you for pointing this out. We have corrected it accordingly.  

**5) Choice of the hosing region** *(Section3.1, paragraph 3) “The freshwater hosing is applied between 20°N and 50°N across the full width of the Atlantic (Figure S3- See underlying data) to reproduce the hosing region by Rahmstorf et al.[27].” Could you comment on the influence of this choice? Different hosing areas have since then been compared, for instance in [1].* We selected this region to apply the hosing in cGENIE because it is the one used in previous hosing experiments with the same model (see Rahmstorf et al. (2005)2 and Gérard and Crucifix (2025)). The rationale behind choosing this specific latitude band was to deliberately avoid deep convection regions, in order not to shut down the AMOC too abruptly. Furthermore, Laridon (2024)3 has shown that the differences between this latitude band and a higher one, such as 50°N–70°N, are not significant, given that cGENIE is not a high-resolution model. What matters most when selecting the hosing region in the Atlantic is the consistency of its location across the different complex models that we aim to emulate. This ensures that no indirect bias is introduced in the subsequent calibration and comparison of the AMOC emulator versions.  

 2 Rahmstorf S, Crucifix M, Ganopolski A, Goosse H, Kamenkovich I, Knutti R, et al. Thermohaline circulation hysteresis: A model intercomparison. Geophysical Research Letters [Internet]. 2005 [cited 2023 Oct 22];32(23). Available from: https://onlinelibrary.wiley.com/doi/abs/10.1029/2005GL023655   3 Laridon Amaury. Development of a Simplified Dynamics Emulator and Investigation of Cascading Collapses of the AMOC and Greenland Ice Sheet in a Climate Model [Internet]. UCLouvain; 2024. Available from: https://dial.uclouvain.be/memoire/ucl/object/thesis:46552  

**6) Drop in the AMOC intensity after 3000 years** *(Section 3.2) “However, after 3,000 years, a sudden drop in AMOC intensity, followed by a partial recovery, is not captured by the ATCM.” ○ Do you know why such drop happens? Had it already been observed?* J. Gérard, one of the co-authors of this paper and the researcher who conducted the cGENIE simulations, has substantial expertise with this model, which he also employs in his own research. According to his interpretation, the abrupt decrease observed likely corresponds to the AMOC entering the self-sustained oscillatory regime that exists in cGENIE, as previously described in Gérard and Crucifix (2024) 1 (see Figure 7a). However, in our setup, the AMOC is forced not only by atmospheric temperature anomalies but also by freshwater fluxes, which further limits the time the system remains within this limit cycle. As a result, the system exits the cycle rapidly, preventing a full oscillation from becoming apparent. This behaviour is therefore understood to be specific to the cGENIE model.  

 1 Gérard J, Crucifix M. Diagnosing the causes of AMOC slowdown in a coupled model: a cautionary tale. Earth System Dynamics. 2024 Mar 22;15(2):293–306.  

**7) References of the design of the emissions scenarios ?** *(Section 3.4) “As an application, we studied the response of the ATCM once integrated into SURFER under 15 different emission scenarios spanning SSP1-2.6 to SSP5-8.5.” ○ Do you have any references on the design of these scenarios?* Indeed, we had not specified the references for these scenarios. The reader can find them in Appendix A.2 of V. Couplet’s PhD thesis. We have now added the corresponding details in the manuscript, as well as in the “Data Availability” section.  

**8) Typo of references in Figure 5** *At the end of Section 3.3 and in Section 3.4, you reference Figure 5 twice, although it should be Figure 6.* Yes, indeed — thank you for pointing this out. We have corrected it accordingly.  

**9) Typo in Supplementary Materials** *(Supplementary Material, Section 1)“We denote* *��������* *, in the case of the GIS as the first calibration experiment of the GIS volume in relation to temperature.” ○ This sentence should be rephrased for clarity.* We have revised this sentence to make it clearer.  

**10) Typo in Supplementary Materials** *(Supplementary Material, section 2) “The duration of* *��������* *was choose to” ○ The verb should be “chosen”.* Yes, indeed — thank you for pointing this out. We have corrected it accordingly.  

**11) Typo in Supplementary Materials** *(Supplementary Material, Section 4) “For* *��������* *, it was also a 20,000-year run,” This is not consistent with the beginning with the rest of the paragraph. From what I understand from the end of the paragraph, the full EXPB run lasts 20,000 years. But the EXPA run lasts 40,000 years. So in the sentence quoted here, you cannot use “also” and “20,000 years” together, otherwise it’s very confusing. In this same sentence, “-0.2 Sv” is cut, with “-“ on one line and “0.2 Sv” on the next, although they should stay together for clarity.* The issue has been resolved by removing Section 4 from the Supplementary Materials, for the reasons previously mentioned. This section was indeed particularly confusing and did not add any substantial or relevant content to the overall presentation.
